# Structural and stratigraphic controls on reservoir distribution in the Ptah oil field, Shushan Basin, North Western Desert, Egypt

**DOI:** 10.1038/s41598-026-52468-w

**Published:** 2026-06-10

**Authors:** Khaled EL Sayed Shamia, Adel Ali Ali Othman, Mohamed Fathy

**Affiliations:** Department of Geology, Faculty of Science, Al_Azhar University, Nasr city, Egypt

**Keywords:** Shushan Basin, Ptah field, Seismic interpretation, Structural traps, Petrophysical analysis, Petrographic analysis, Reservoir characterization, Integrated interpretation, Environmental sciences, Solid Earth sciences

## Abstract

The Ptah Oil Field, located along the northwestern margin of the Shushan Basin in Egypt’s Western Desert, hosts structurally complex clastic reservoirs whose hydrocarbon distribution is strongly fault controlled. This study applies an integrated seismic–petrophysical–petrographic workflow to characterize reservoir architecture and trapping mechanisms within the Bahariya, Lower Cretaceous Alam El Bueib (AEB-3D/3E) and Paleozoic Shiffah B sandstone intervals. Interpretation of 20 two-dimensional (2D) seismic lines extracted from 3D seismic cube, calibrated with well logs and core from six wells, reveals an extensional fault framework dominated by NW–SE and E–W normal faults forming horst–graben geometries and three-way dip closures that govern reservoir preservation and compartmentalization. Petrophysical analysis indicates moderate reservoir quality with significant lateral variability in reservoir properties. The net pay thickness varies across the study area and reached to 13, 38 and 129 ft. for Bahariya, Alam El Bueib and Paleozoic Shiffah B Sandstone reservoirs. Effective porosity derived from well logs ranges approximately between 7–12%, while core measurements indicate porosity values reaching 21%. Permeability values obtained from core analysis vary widely between 0.02 and 839 mD, reflecting heterogeneous pore systems within the sandstone reservoirs. Structural mapping identifies several fault-bounded closures with aerial extents ranging from approximately 273 to 4500 acres across the studied stratigraphic intervals, these structural configurations may represent promising exploration targets, particularly where fault sealing and reservoir quality are favorable. Petrographic observations confirm that diagenetic clay distribution further controls pore structure and permeability. Integrated results demonstrate that hydrocarbon accumulation is primarily dictated by the interaction of fault architecture, stratigraphic preservation, and diagenetic modification rather than reservoir properties alone. This framework refines reservoir delineation, reduces exploration uncertainty, and identifies structurally favorable targets, providing a transferable methodology for evaluating fault-controlled clastic reservoirs in extensional basin settings.

## Introduction

**Fig. 1:**
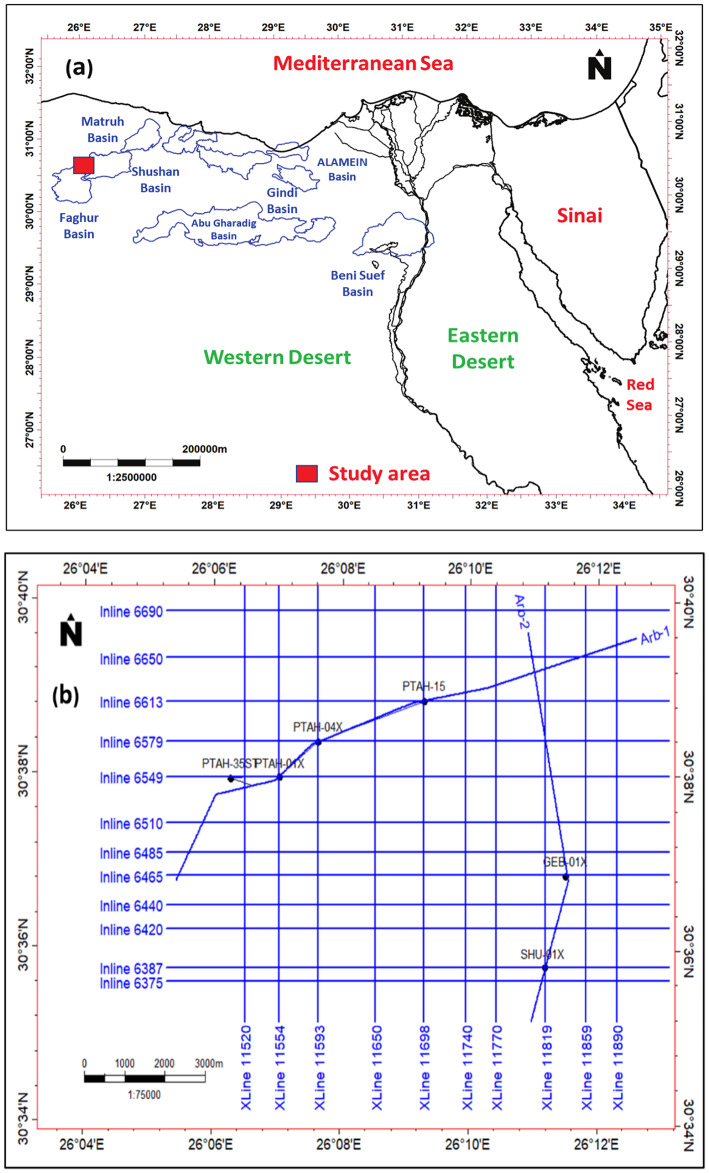
**a**) Egypt’s geographic map with the location of study area modified after^1^. **b**) Location map showing seismic lines and available wells in the study area, generated using SLB Petrel software, Version 2018.

**Fig. 2:**
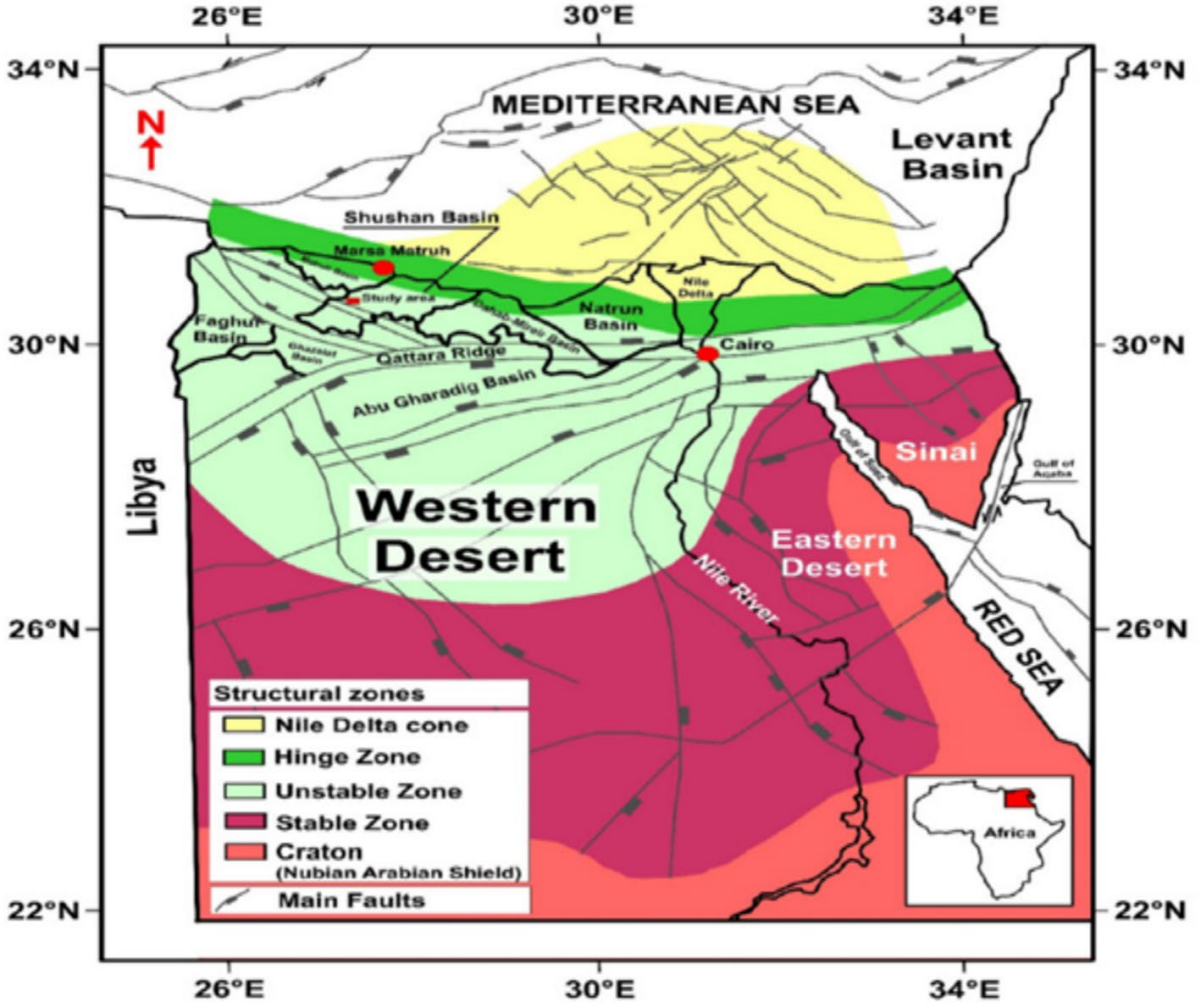
The main structural zones projected on a regional map of Egypt, modified after^8–13^.

Egypt’s Western Desert is the country’s largest hydrocarbon-producing province. Rift-related extensional basins in the Western Desert of Egypt host several significant hydrocarbon accumulations, including those within the Shushan Basin where the Ptah Field is located^1–5^. The Ptah Field lies along the northwestern margin of the Shushan Basin, a key exploration area producing hydrocarbons from Paleozoic, Alam El Bueib, and Bahariya reservoirs^6–9^. The study area covers approximately 112 km^2^ between latitudes 30°04′–30°35′ N and longitudes 26°05′–26°13′ E as shown in (Fig. 1). The Shushan Basin forms part of the Northern Western Desert within the Unstable Zone as shown in (Fig. 2) is characterized by thick sedimentary successions influenced by Late Cretaceous–Early Tertiary tectonic activity^9–14^. Syrian Arc deformation and associated fault systems generated complex half-graben geometries that control reservoir preservation, migration pathways, and trap development^15^. Hydrocarbon accumulation reflects a favorable combination of source rocks, sandstone reservoirs, and shale seals, with fault architecture playing a dominant role in trap formation and fluid migration^16–19^. The main reservoirs considered in this study are the Bahariya, Lower Cretaceous Alam El Bueib Formation (AEB-3D/3E) units and the Paleozoic Shiffah B Sand, all exhibiting lateral heterogeneity and variable reservoir quality. Several previous studies have investigated the petroleum systems and reservoir characteristics of the Ptah Field. For example,^16^ focused on seismic stratigraphy, petrophysical rock typing, and organic geochemistry to understand the controls on hydrocarbon accumulation.^17^ evaluated hydrocarbon potential in the Paleozoic reservoir using petrophysical analysis and pressure gradient interpretation. While^18^ concentrated on determining Archie petrophysical exponents for the Alam El Bueib reservoir using Pickett plot analysis. In contrast, the present study integrates seismic structural interpretation, petrophysical property distribution, and petrographic observations to investigate the structural controls on reservoir distribution in the Ptah Field. This approach establishes a tectono-stratigraphic framework linking fault architecture with reservoir compartmentalization and hydrocarbon trapping, providing improved insights into reservoir architecture and identifying structurally favorable targets for future exploration and drilling. Despite previous investigations addressing seismic stratigraphy and petrophysical characteristics in the Ptah Field, the relationship between fault architecture and spatial reservoir distribution remains insufficiently constrained. In particular, previous studies focused either on seismic stratigraphic interpretation or isolated petrophysical evaluation of individual reservoirs. The present study addresses this gap by integrating seismic structural interpretation with spatial petrophysical mapping and petrographic observations for different reservoirs including Bahariya, Alam EL Bueib and Paleozoic Shiffah B Sand reservoirs. This integrated workflow allows evaluation of how fault geometry, structural closure development, and lithological heterogeneity interact to control reservoir compartmentalization and hydrocarbon accumulation across multiple stratigraphic levels. The results provide new insights into structurally controlled reservoir distribution in the Ptah Field and offer a transferable methodology for evaluating fault-controlled clastic reservoirs in extensional basin settings. This integration helps identify structurally favourable zones that may represent potential drilling targets. These zones are primarily associated with structurally elevated fault-bounded closures where reservoir quality and hydrocarbon saturation coincide, providing guidance for future exploration and development activities.

## Geologic setting

### Regional tectonic framework

The tectonic evolution of the Shushan Basin reflects a complex interaction between Paleozoic sedimentation, Mesozoic extensional tectonics, and later compressional deformation associated with the Syrian Arc system. During the Paleozoic, relatively stable tectonic conditions allowed the deposition of thick clastic sequences, including the Shiffah B sandstone units that form an important reservoir interval within the Ptah Field. This phase was followed by regional uplift and erosion, resulting in the development of an erosional unconformity that locally truncates the Paleozoic succession and influences reservoir continuity and thickness distribution. During the Jurassic to Early Cretaceous, the northern Western Desert experienced regional extensional tectonics related to the opening of the Neo-Tethys. This extensional regime generated a system of normal faults and half-graben structures that controlled sediment accommodation, stratigraphic thickness distribution, and facies variability across the basin^19,20^. Many of the major structural elements observed in the Ptah Field, including tilted fault blocks and horst–graben geometries, were initiated during this phase. Importantly, these fault systems developed prior to or contemporaneously with hydrocarbon migration, providing effective migration pathways and structural traps. Later, during the Late Cretaceous to Early Tertiary, compressional deformation associated with the Syrian Arc tectonic system resulted in partial reactivation of pre-existing extensional faults and structural inversion. This tectonic reactivation enhanced structural relief, improved trap integrity, and contributed to the formation of fault-bounded closures. The timing of this deformation relative to hydrocarbon generation further enhanced trapping efficiency within structurally elevated compartments. Hydrocarbon generation is mainly attributed to deeply buried Paleozoic source rocks, with migration occurring along major fault systems and permeable carrier beds toward structurally elevated blocks. As a result, the present structural configuration of the Ptah Field reflects the combined influence of early extensional faulting and later tectonic reactivation, which together controlled reservoir preservation, migration pathways, and hydrocarbon accumulation within the basin.

### Stratigraphy of the shushan basin

**Fig. 3:**
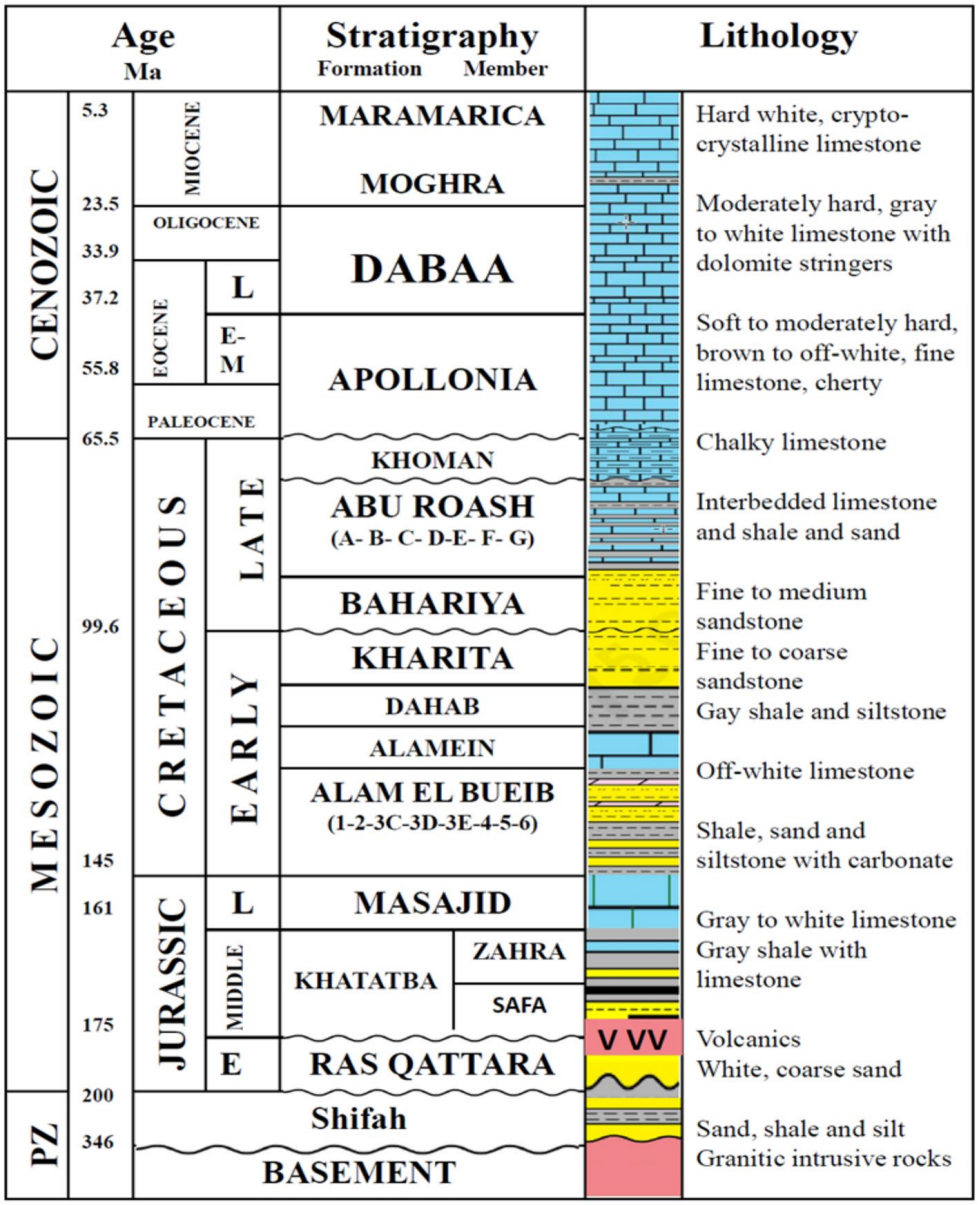
General stratigraphy of the Unstable Zone of the Northwestern Desert, Egypt, modified from^1,20,21^.

The basin stratigraphy ranges from Lower Paleozoic to Recent deposits, with the Bahariya, Alam El Bueib (AEB), and Shiffah Formations representing the principal hydrocarbon-bearing intervals (Fig. 3).

**Fig. 4:**
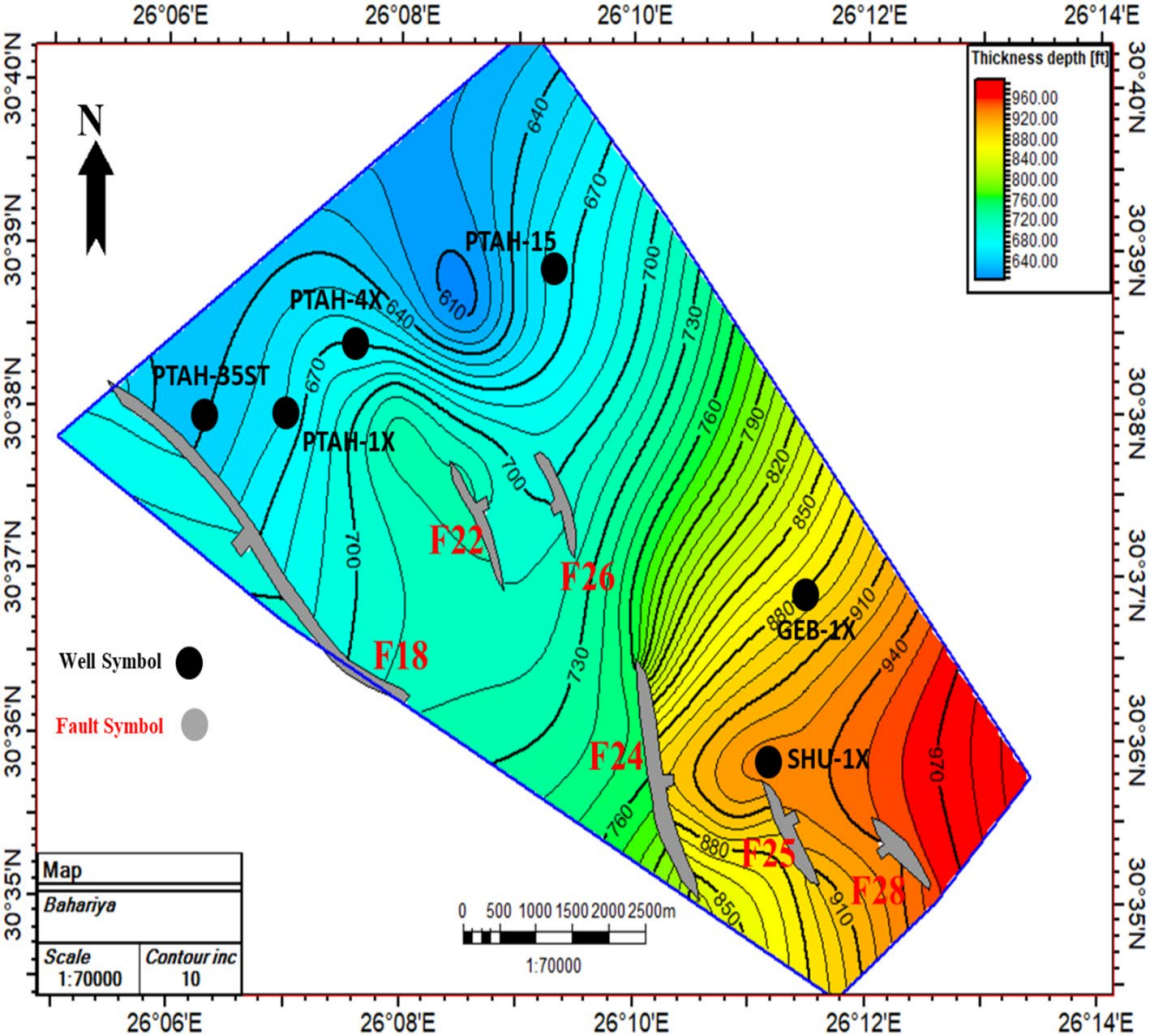
Thickness variation map for Bahariya Formation in Ptah field.

**Fig. 5:**
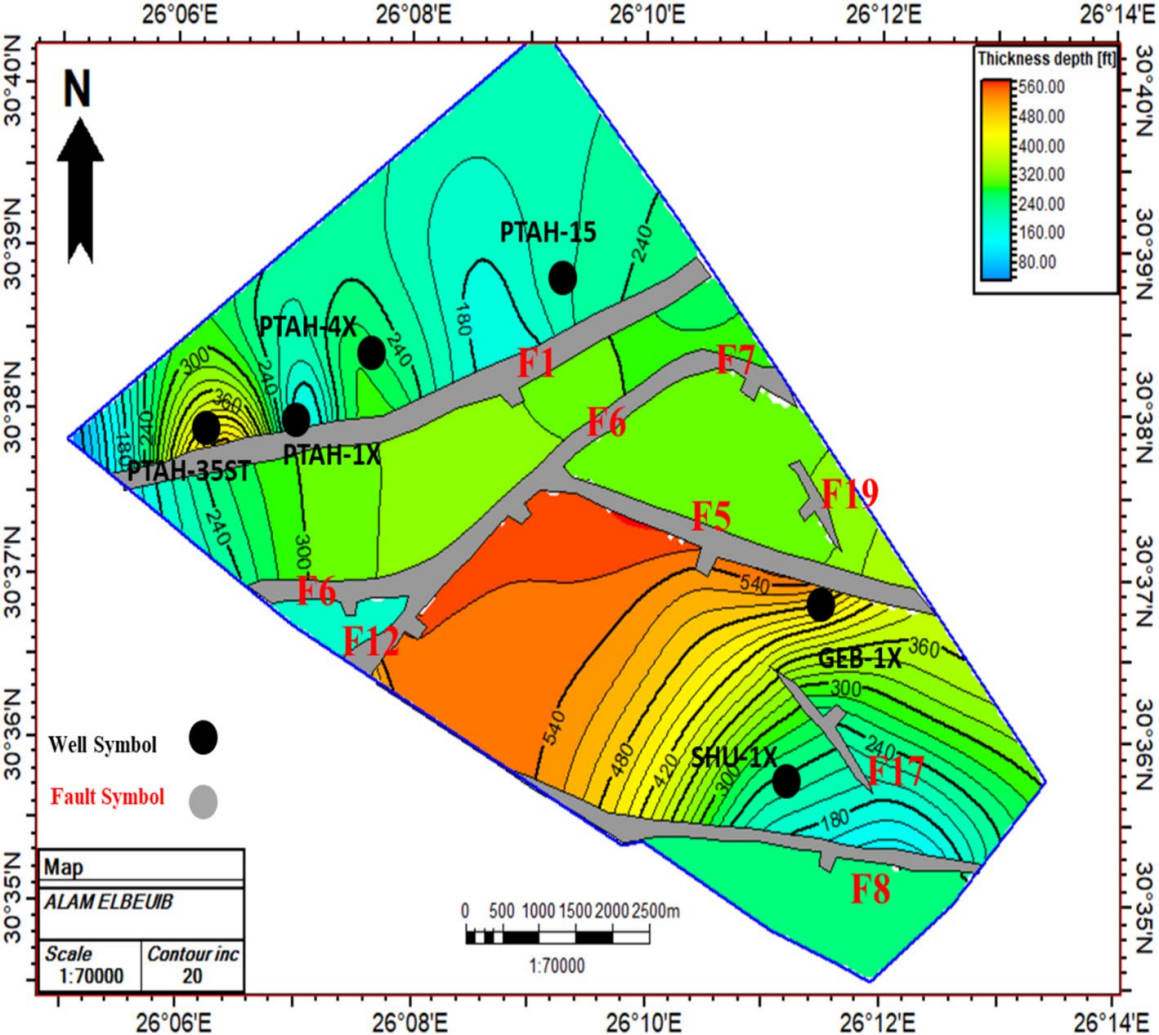
Thickness variation map for AEB-3D/3E unit in Ptah field.

**Fig. 6:**
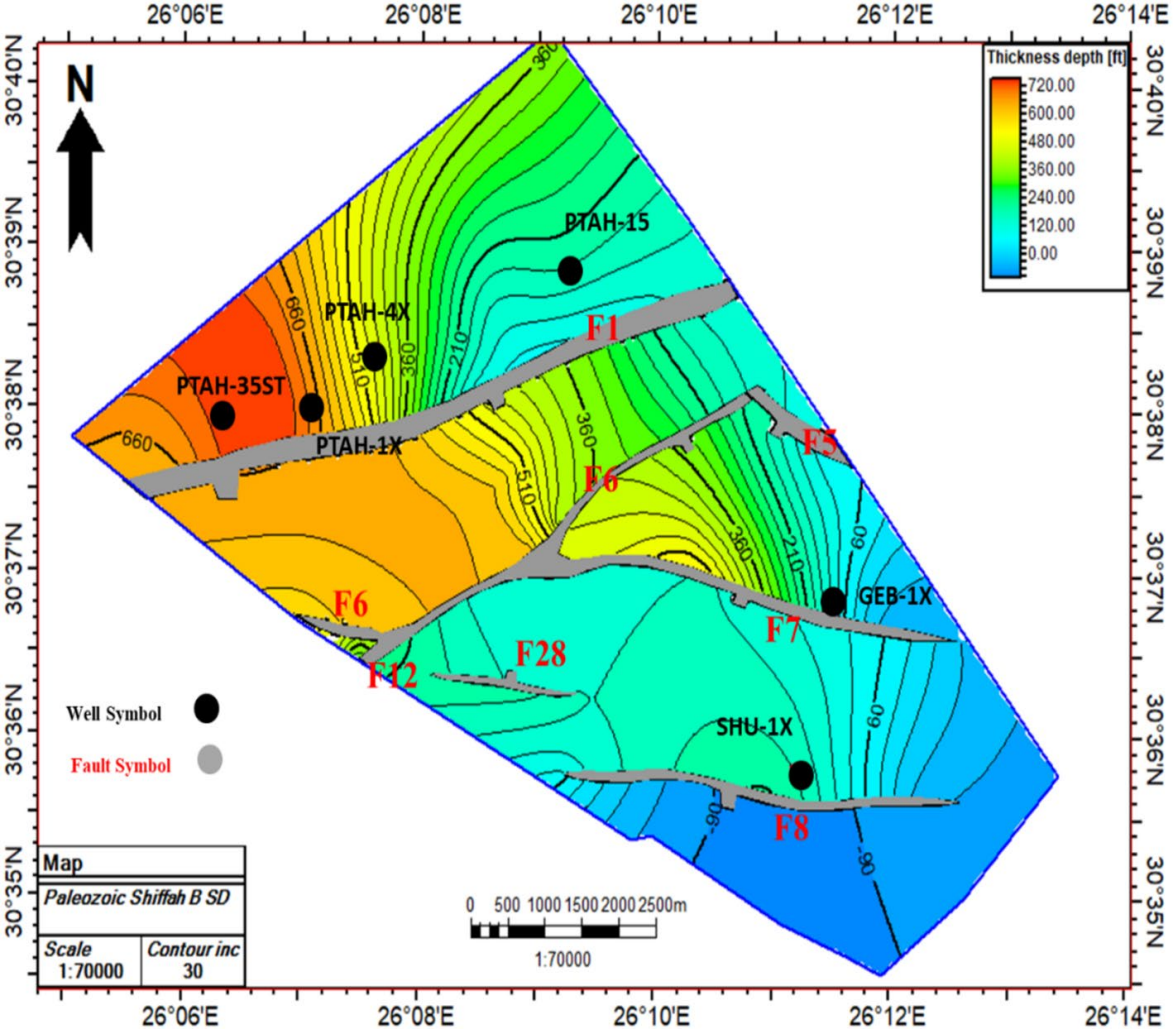
Thickness variation map for Paleozoic Shifah B sand unit in Ptah field.

The Cenomanian Bahariya Formation consists predominantly of quartzose sandstones deposited in fluvio-deltaic to shallow-marine environments^6,15,20–22^. Thickness variations for Bahariya across the study area and thickening towards south east direction reflect depositional trends and structural influences (Fig. 4). The Lower Cretaceous Alam El Bueib Formation includes several subdivisions, of which the AEB-3 interval is further divided into multiple subunits. In this study, emphasis is placed on the AEB-3D and AEB-3E units, which represent the main reservoir intervals characterized by laterally variable sandstone bodies deposited under shallow-marine to fluvio-deltaic conditions^23,24^. Thickness distribution indicates increasing towards the center of the study area (Fig. 5). The Paleozoic succession of the Shushan Basin includes multiple formations such as Shiffah, Kohla, Basur, Zeitoun, Desouqy, Dhiffah, and Safi. The Shiffah B Sandstone is characterized by medium- to coarse-grained sandstones interbedded with siltstone and minor shale^25^. Thickness variations, with increasing thickness toward the northwest direction, reflect regional structural influences (Fig. 6), and fault-related geometries affect reservoir connectivity and migration pathways.

### Petroleum system

The petroleum system in the Shushan Basin is characterized by the interaction of Paleozoic to Mesozoic source rocks, clastic sandstone reservoirs, and regional shale seals^2,16^. Hydrocarbon generation is mainly attributed to organic-rich source rocks within the deeper Paleozoic succession of the basin^2,5^. Migration of hydrocarbons likely occurred along major normal fault systems and permeable carrier beds toward structurally elevated blocks. Reservoir rocks are mainly represented by the Bahariya, Lower Cretaceous Alam El Bueib sandstones and the Paleozoic Shiffah B sandstone units, which exhibit variable reservoir quality. Effective sealing in the Ptah Field is provided by shale-dominated intervals within the Bahariya Formation and the Alam El Bueib Formation. In the Bahariya reservoir, sealing occurs where the reservoir is juxtaposed against the Bahariya shale. Similarly, the Alam El Bueib-3D/3E reservoirs are sealed where they are juxtaposed against the Alam El Bueib-3C shale. In the deeper section, the Paleozoic Shiffah B sandstone reservoir is sealed by juxtaposition against the Alam El Bueib-6 shale. Structural traps associated with normal faulting and horst–graben geometries constitute the primary hydrocarbon trapping mechanism in the Ptah Field.

## Materials and methods

### Data sources and analytical workflow

**Fig. 7:**
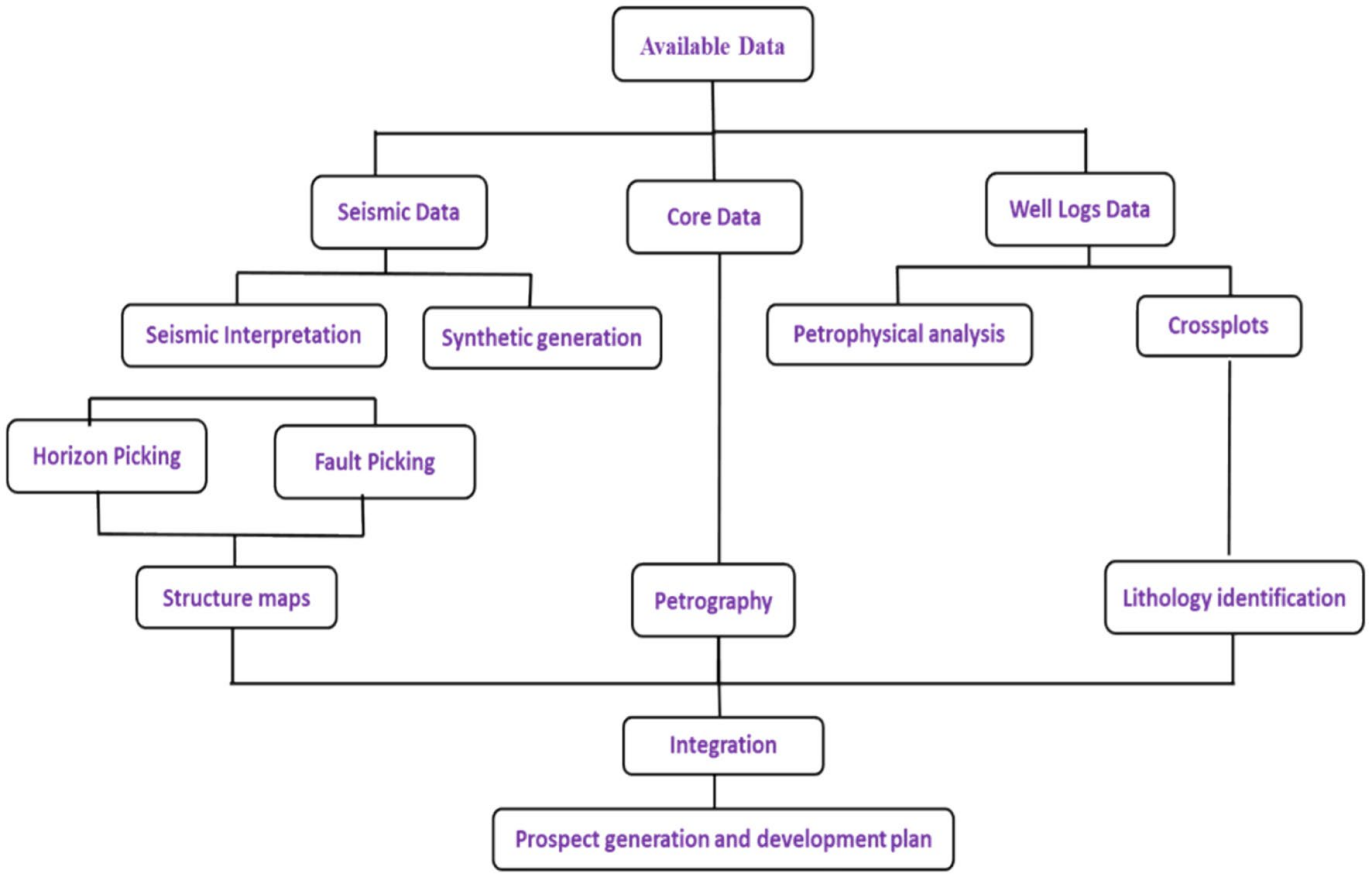
Flowchart showing the work flow used in the study.

This study integrates seismic interpretation, petrophysical analysis, and petrographic core evaluation to characterize reservoir architecture and structural controls within the Ptah Field. All datasets were provided by Khalda Petroleum Company (KPC). The analytical workflow follows a sequential framework consisting of seismic interpretation and well-to-seismic calibration, petrophysical evaluation, petrographic characterization, and integrated interpretation (Fig. 7).

### Seismic data interpretation

The seismic dataset comprises twenty 2D post-stack seismic lines covering the study area extracted from 3D seismic cube (Fig. 1). The seismic line spacing is sufficient to identify major fault systems and structural closures within the study area and ranges between (500-1000 meter). The dominant frequency of the seismic data ranges approximately between 20–80 Hz, which allows reliable imaging of major structural features. The data have a recording window of 6.0 s two-way travel time (TWT) and a sampling interval of 4 ms.

### Seismic resolution and bed thickness

Seismic resolution plays a critical role in stratigraphic analysis, as the ability to distinguish subsurface layers is largely controlled by the resolving power of the seismic data. Resolution can be classified into vertical and horizontal components. The resolving capability of seismic reflections depends primarily on the frequency and wavelength of the seismic signal. Higher-frequency waves, characterized by shorter wavelengths, provide improved resolution but are more susceptible to attenuation within the subsurface. In contrast, lower-frequency waves, with longer wavelengths, penetrate deeper into the subsurface but offer reduced resolution.^26^ investigated thin sandstone beds in Northwest China and demonstrated that amplitude and waveform analysis, combined with forward modeling, can be used to identify pinch-out boundaries through the seismic tuning effect. Their results indicate that bed thicknesses less than one-quarter of the dominant wavelength (λ/4) can still be detected based on amplitude variations. In the present study, both vertical and horizontal seismic resolution concepts are applied to evaluate the detectability of thin stratigraphic units within the study area.

#### Vertical resolution

It is defined as a measure of how large an object needs to be in order to be seen on a vertical seismic section. It is calculated as a quarter wavelength and layers may be detected when their thickness is below this value. The wavelength is calculated by *λ* = *V*/*F* and the vertical seismic resolution is calculated by *λ*/4 = *V*/4*F*^27^, where *λ* = wavelength, *V* = seismic velocity, and *F* = seismic frequency. The seismic data are processed to zero-phase and displayed with reverse polarity.

#### Horizontal resolution

Horizontal resolution is controlled by the Fresnel zone, which represents the portion of the reflector contributing to the recorded signal at a given depth, and is calculated as follows: Fresnel zone before migration = V ${\left(T/F\right)}^{0.5}$, Fresnel zone after migration = *λ*/4 = V/4*F*^27^, where; *λ* = wavelength, *V* = seismic velocity, *F* = seismic frequency, and *T* = depth in time. In the subsurface, all features with lateral extent greater than the Fresnel zone will be visible. An object can be seen on seismic if it is larger than either the vertical or horizontal resolution. the dominant frequency of the 3D-seismic cube is about 50 Hz, and interval velocity is 13500 ft./s, then seismic resolution= 13500 / (4 × 50) = 67.5 ft. The Bahariya sandstone, with thicknesses generally below 67.5 ft, is therefore likely to be below seismic resolution and influenced by tuning effects. In contrast, the AEB-3D and Shiffah units, with thicknesses ranging from 60 to 150 ft, are partially resolvable; thicker intervals (>67.5 ft.) can be detected as separate reflectors, while thinner portions may still be affected by tuning.

Seismic interpretation was conducted using Schlumberger Petrel software (2018 version) (https://www.software.slb.com/products/petrel). Although 3D seismic data provide higher resolution, the available 2D seismic grid provides adequate structural coverage to interpret the main fault trends and structural closures controlling reservoir distribution in the Ptah Field.

### Well-to-seismic calibration

Well-to-seismic calibration was achieved through the generation of a synthetic seismogram using calibrated density (RHOB) and sonic (DT) logs from the Ptah-4X well. The logs were conditioned and used to derive P-wave velocity, from which acoustic impedance was calculated. Reflection coefficients were computed based on impedance contrasts. A representative zero-phase wavelet was extracted from seismic data in the vicinity of the well, and the reflection coefficient series was convolved with the extracted wavelet to produce the synthetic trace, enabling accurate well-to-seismic tie and horizon identification.

### Horizon and fault interpretation

Key stratigraphic horizons, including the Bahariya, Alamein, Alam El Bueib-3D, and the Paleozoic Shiffah Formations, as well as major fault systems, were interpreted across the seismic grid. Although the seismic interpretation is presented along selected 2D sections, the data are derived from a 3D seismic volume. Accordingly, the variance attribute was computed from the 3D dataset and used to enhance fault delineation in map view.

### Time-to-Depth conversion

Time-structure maps were constructed for each horizon and subsequently converted to depth using average velocity following standard seismic conversion principles^28^. These procedures enabled structural framework mapping and identification of fault geometries. Although minor uncertainties may arise from velocity variations between wells and seismic resolution, the resulting depth maps are considered reliable for first-order structural interpretation. Based on well-to-seismic calibration and typical uncertainties associated with 2D seismic data, the estimated depth uncertainty is approximately ±30 ft, which does not significantly affect the identification of the major structural closures within the study area.

### Well-Log petrophysical analysis

Petrophysical evaluation was performed using a complete suite of wireline logs from six wells summarized in (Table 1), all penetrating Paleozoic intervals. All wells include the essential log types required for petrophysical evaluation, including gamma ray, density, neutron, sonic, and resistivity logs. Interpretation was conducted using Schlumberger Interactive Petrophysics (IP) software, version 4.5 (https://www.software.slb.com). Lithological discrimination employed standard crossplot techniques following established methodologies^10,29^.

**Table 1:** Available and used well-logs data.

**Well name**	**Total depth Md(ft.)**	**Available data**						
		**Gamma ray**	**Resistivity**	**Density**	**Neutron**	**Photoelectric factor**	**Sonic**	**Check shot**
**PTAH-01X**	**13800**	✔	✔	✔	✔	✔	✔	✔
**PTAH-04X**	**13160**	✔	✔	✔	✔	✔	✔	✔
**PTAH-15**	**12800**	✔	✔	✔	✔	✔	×	×
**PTAH-35ST**	**12780**	✔	✔	✔	✔	✔	×	×
**SHU-01X**	**11000**	✔	✔	✔	✔	✔	✔	✔
**GEBU-01X**	**10135**	✔	✔	✔	✔	✔	✔	✔

Neutron–density crossplots were used to distinguish lithology and estimate shale content for the producing horizons in the field including Bahariya, Alam El Bueib-3D and Paleozoic Shiffah B sand reservoirs. Reservoir parameters including effective porosity, shale volume, and fluid saturation were evaluated vertically and laterally, supported by iso-parametric mapping to assess spatial variability, and were generated using the inverse distance weighting (IDW) interpolation method based on available well control points. Water saturation was calculated using Archie’s equation. The Archie parameters applied in this study follow standard sandstone reservoir values, where the tortuosity factor (a) = 1, cementation exponent (m) = 2, and saturation exponent (n) = 2. These parameters are consistent with typical sandstone reservoirs in the Western Desert.

### Petrographic and core analysis

Petrographic characterization focused on evaluating mineralogical composition, pore structure, and diagenetic features influencing reservoir quality within the Alam El Bueib and Paleozoic Shiffah intervals. A total of 31 cleaned sidewall core samples from the PTAH-01X well were analyzed. Laboratory investigations (Routine core analysis) were conducted by Corex Company using thin-section petrography, scanning electron microscopy (SEM), and X-ray diffraction (XRD). These analyses supported sandstone classification, identification of diagenetic processes, and assessment of mineralogical controls on porosity and permeability. Petrographic observations were integrated with petrophysical interpretations to validate reservoir heterogeneity and depositional characteristics.

## Results

### Seismic interpretation results

#### Synthetic seismogram calibration

**Fig. 8:**
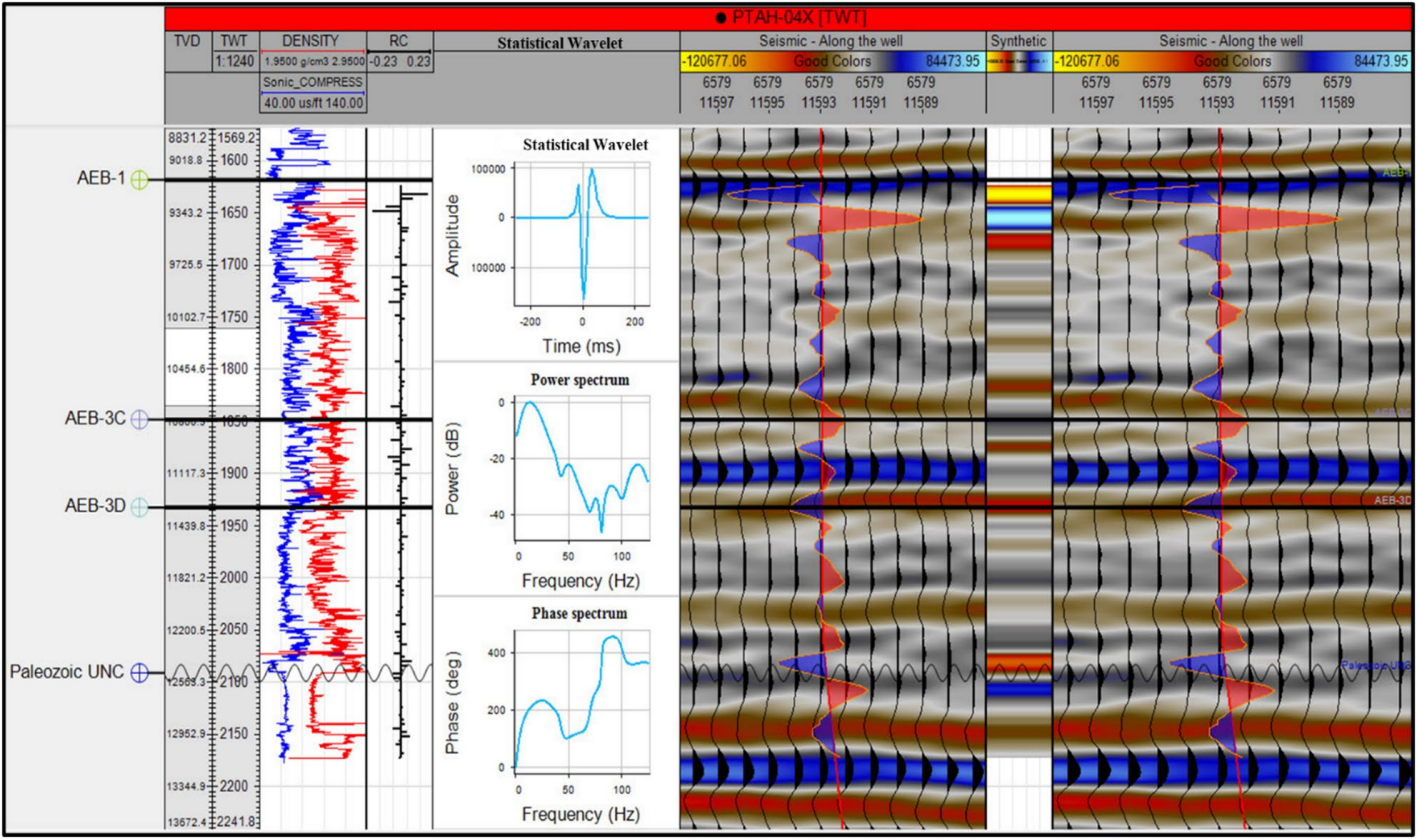
A Synthetic seismogram for well PTAH-4X illustrating correlation between well logs and seismic reflections.

The synthetic seismogram generated from calibrated density and sonic logs of the Ptah-4X well

establishes a reliable tie between well markers and seismic reflections (Fig. 8). The synthetic trace closely matches major seismic events corresponding to the Alam El Bueib, and Paleozoic horizons. Reflection amplitude variations coincide with impedance contrasts at formation tops, confirming consistent horizon identification and time–depth calibration. The time mistie between synthetic and seismic horizons is within acceptable limits (<10 ms.), confirming calibration reliability. This correlation provides a stable reference framework for structural interpretation across the seismic grid.

#### Fault geometry and structural framework

**Fig. 9:**
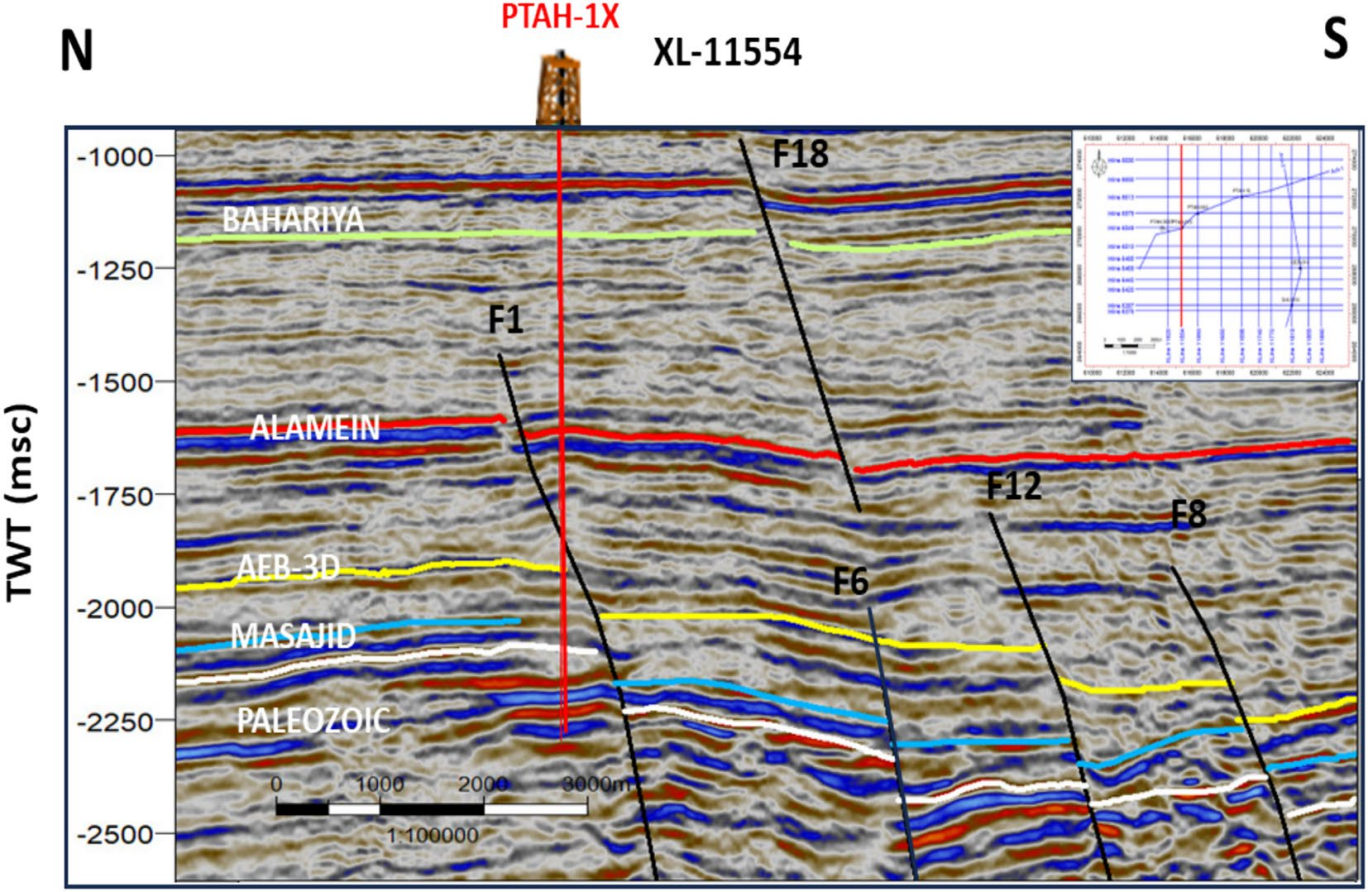
Interpreted seismic section of XL-11554 TWT (msec.), illustrating the area affected by five normal faults.

**Fig. 10:**
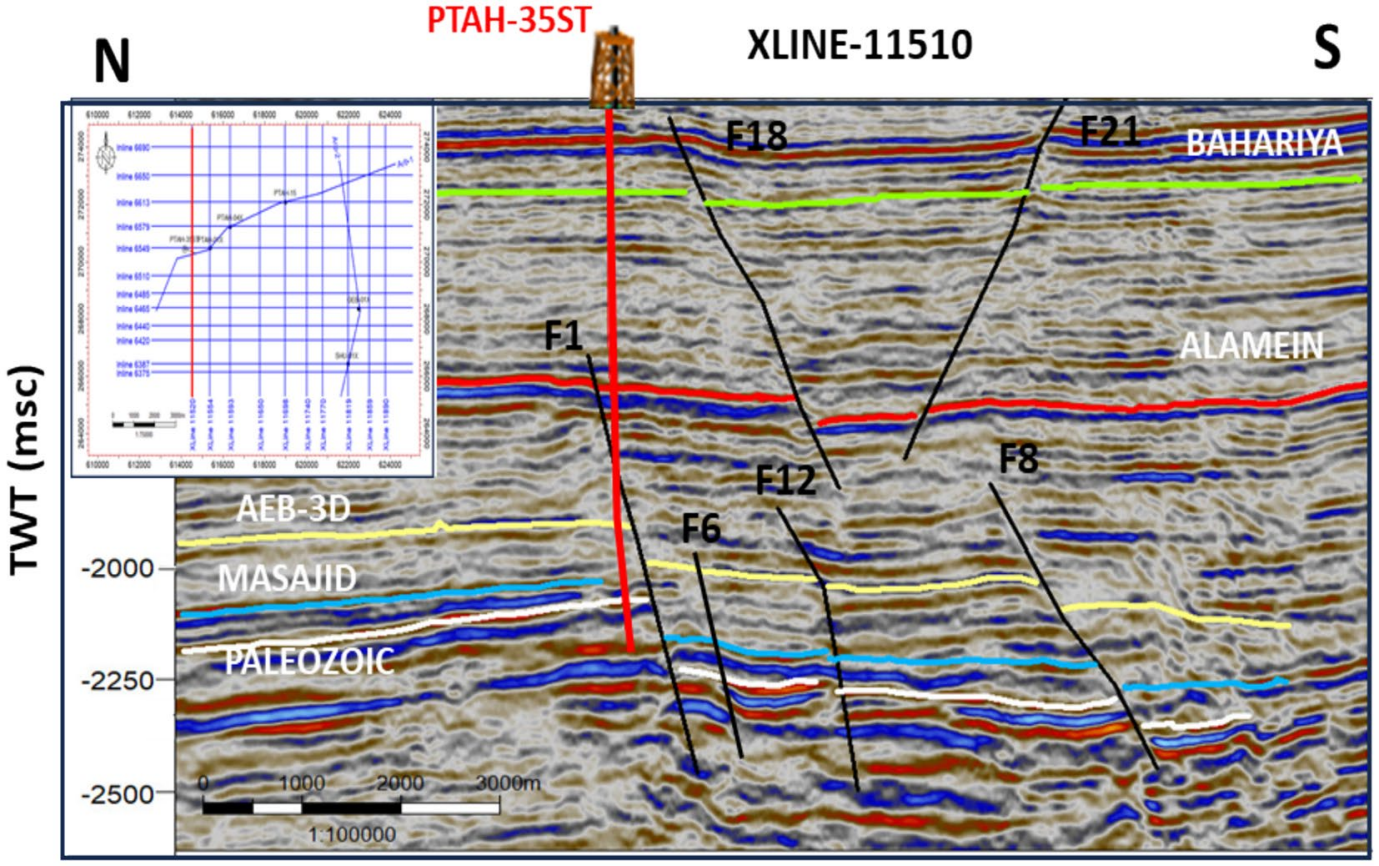
Interpreted seismic section XL-11510 TWT (msec.), illustrating the area affected by six normal faults.

**Fig. 11:**
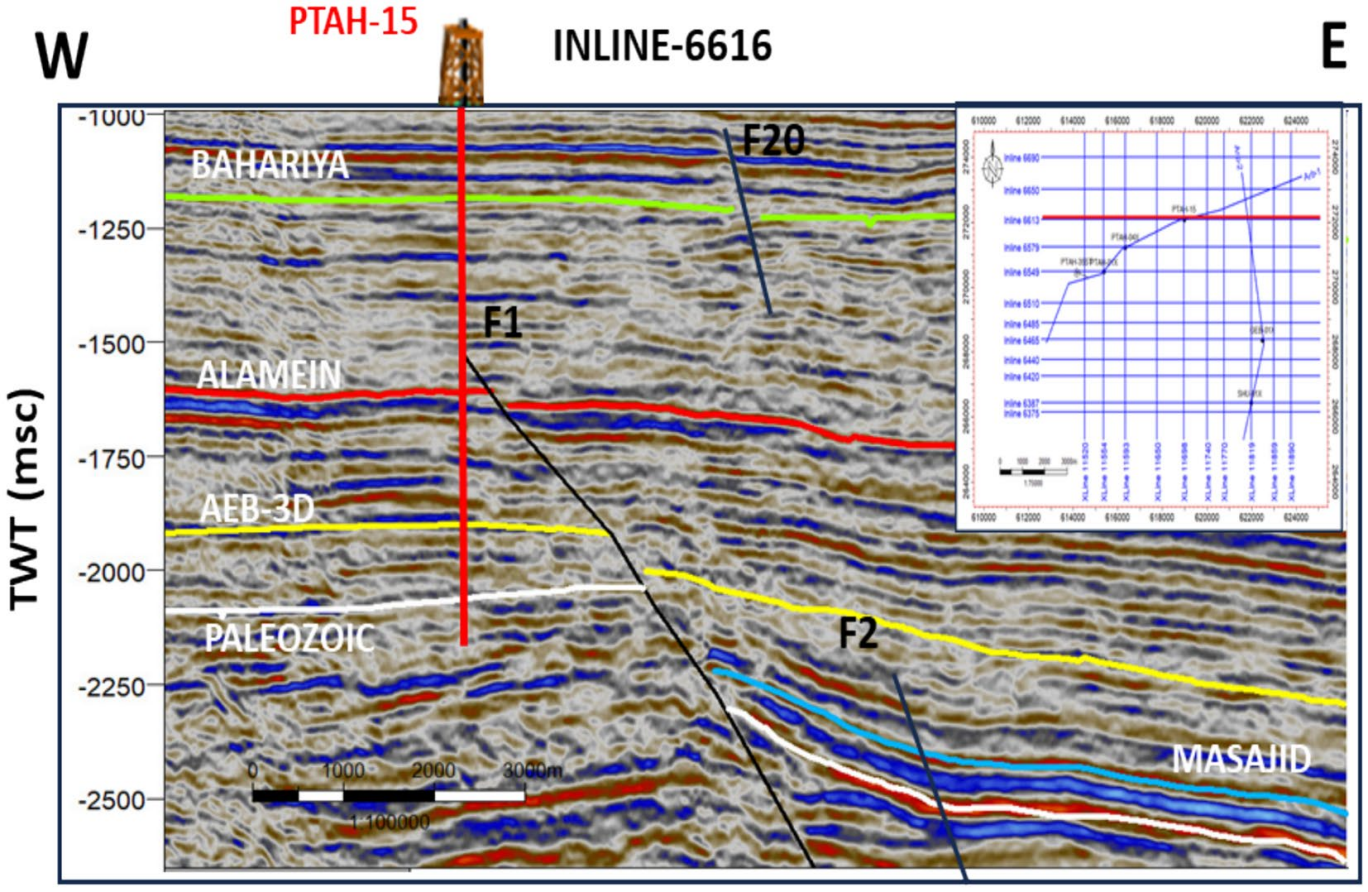
Interpreted seismic section INLINE-6616 TWT (msec.), illustrating the area affected by three normal faults.

**Fig. 12:**
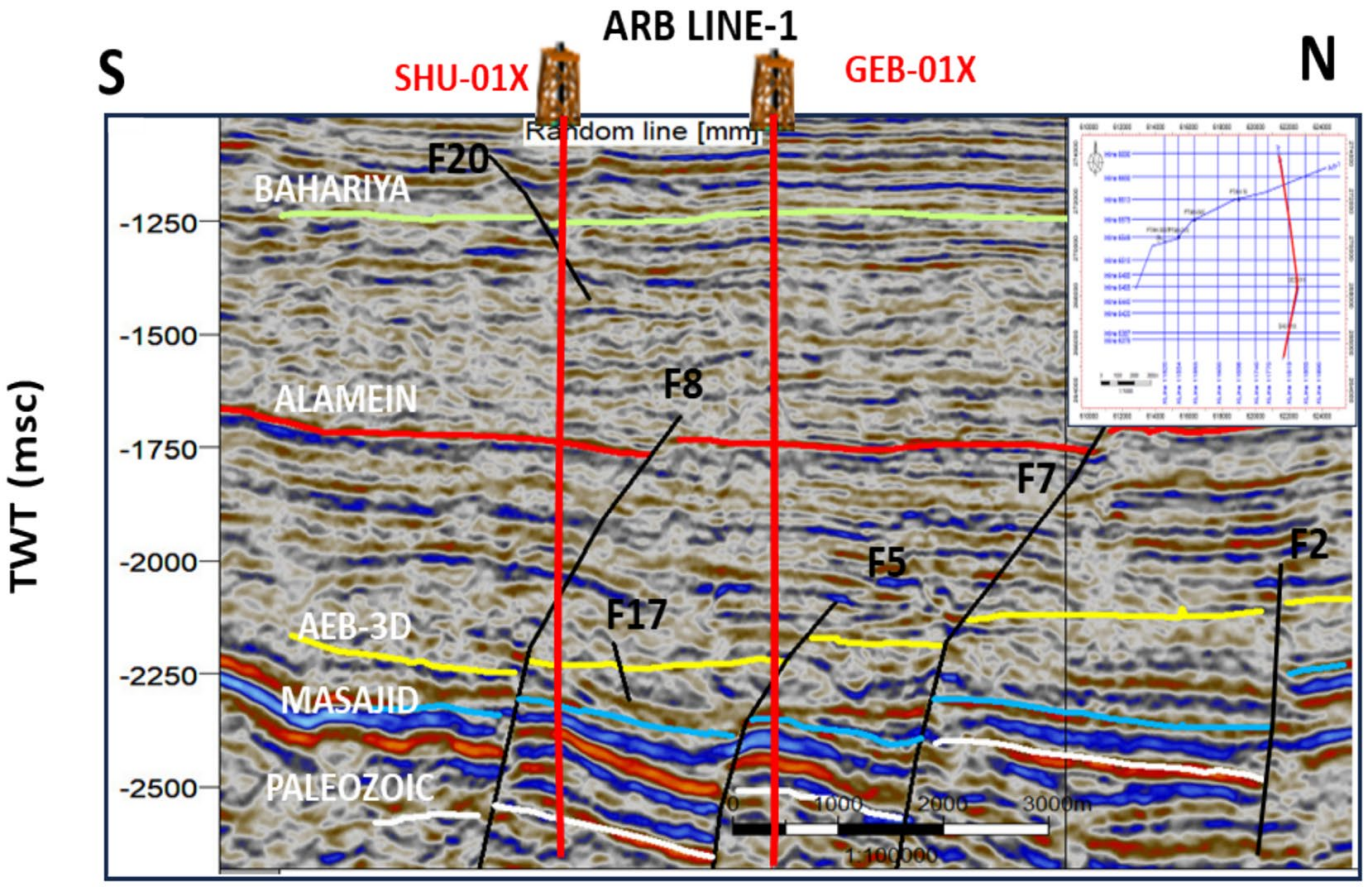
Interpreted seismic section of Arbitrary Line-1 TWT (msec.), illustrating the area affected by six normal faults.

Seismic interpretation indicates that the structural framework of the Ptah Field is dominated by an extensional fault system expressed by two principal fault orientations. Crossline sections (Figs. 9 and 10) show southeast- and northeast-dipping normal faults that offset reflectors corresponding to the Bahariya, Alam El Bueib-3D, and Paleozoic Shiffah B intervals. These faults produce clear vertical separation of reflectors and define tilted fault blocks and localized graben geometries consistent with extensional tectonic models^30,31^. The Crossline sections confirm the continuity of these structures across stratigraphic levels, where reflector packages remain coherent within individual structural blocks but terminate abruptly along major fault planes. Inline seismic section (Fig. 11) illustrates along-strike persistence of the fault network. Major faults penetrate downward into Paleozoic levels, generating consistent reflector displacement, while secondary structures terminate upward within the Bahariya interval. This stratigraphic variation in fault continuity produces stacked structural compartments, a characteristic feature of layered extensional systems^32,33^. Reflector geometry within elevated blocks appears laterally continuous with moderate amplitude contrast, whereas downthrown domains display reflector truncation and localized thinning. Arbitrary seismic section in the eastern sector (Fig. 12) demonstrates similar structural segmentation, where intersecting fault sets partition the stratigraphic section into discrete blocks. Reflector offsets across these faults produce measurable stratigraphic separation, particularly within the Alam El Bueib and Paleozoic intervals, this seismic section highlights the spatial relationship between fault planes and stratigraphic boundaries, showing systematic juxtaposition of reflector packages along fault contacts.

Across all sections, reflector behavior varies with structural position. Elevated blocks display coherent reflector continuity within reservoir intervals, while adjacent downthrown blocks exhibit disrupted or attenuated reflections. Termination patterns along fault planes are consistently observed in crossline, inline, and arbitrary sections (Figs. 9–12), confirming that fault geometry governs reflector distribution and block segmentation.

**Fig. 13:**
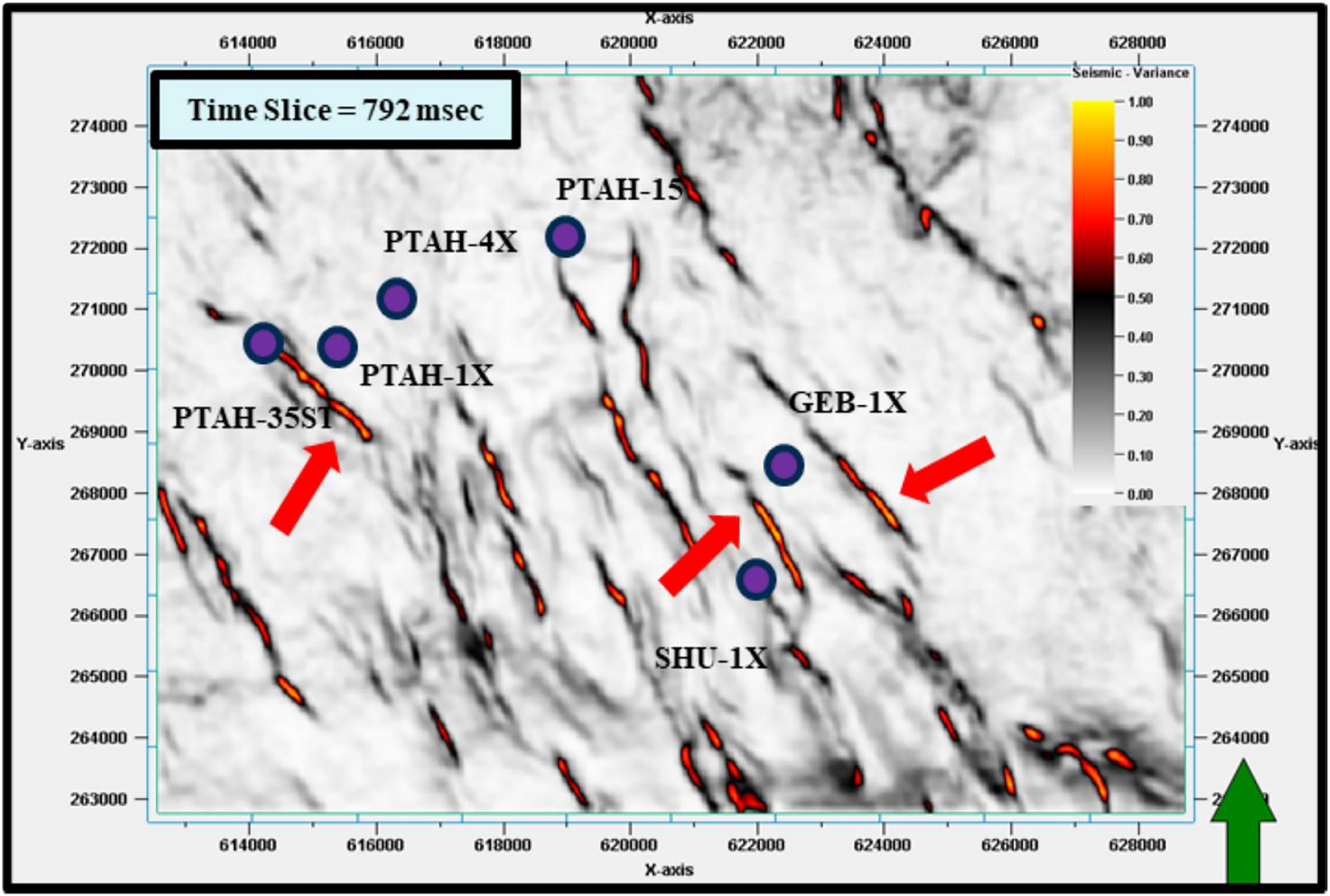
Variance attribute slice at the (792 msec.) corresponding to Bahariya Formation showing structural discontinuities and fault patterns highlighted by red arrows.

**Fig. 14:**
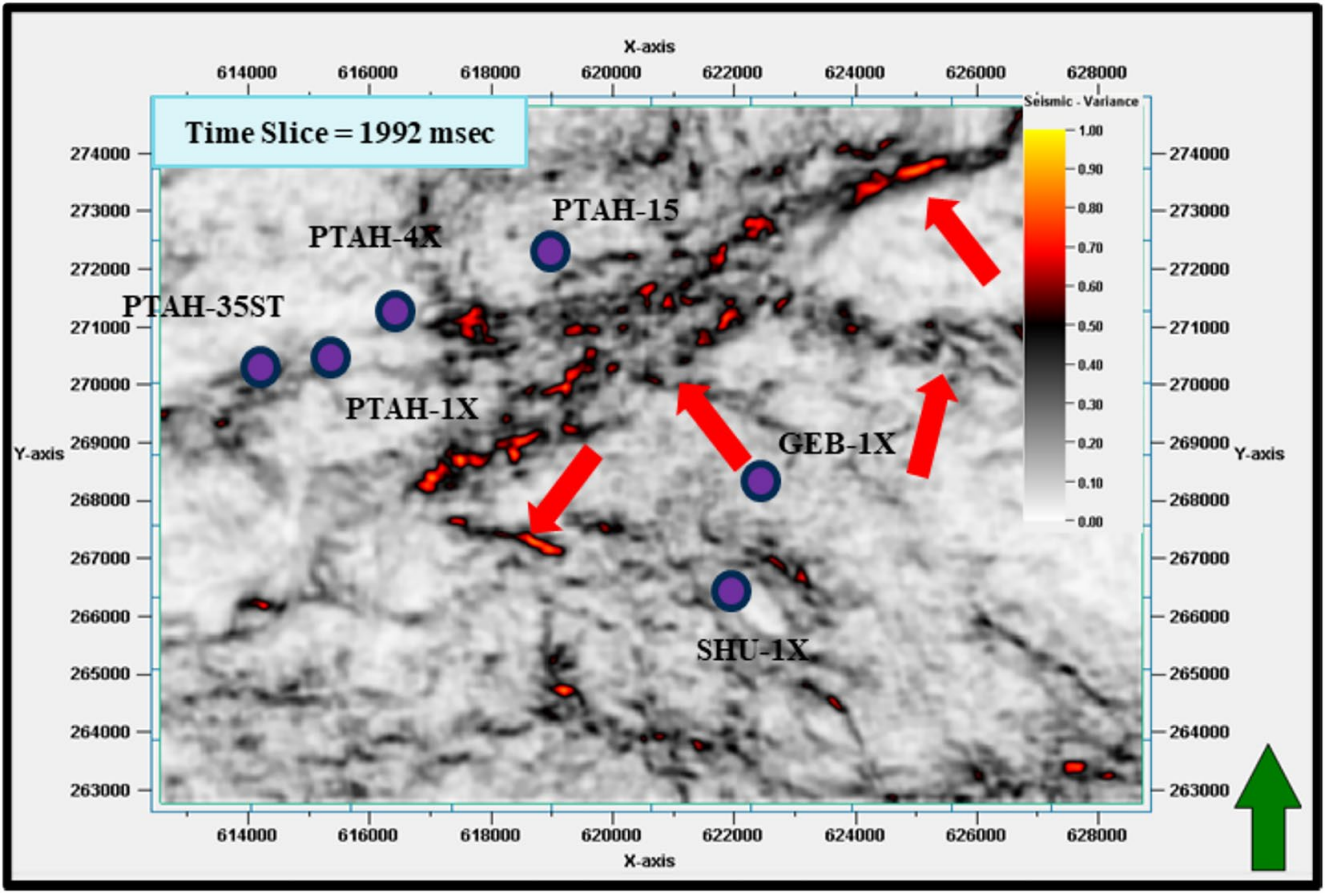
Variance attribute slice at the (1992 msec.) corresponding to Alam El Bueib-3D unit showing structural discontinuities and fault patterns highlighted by red arrows.

**Fig. 15:**
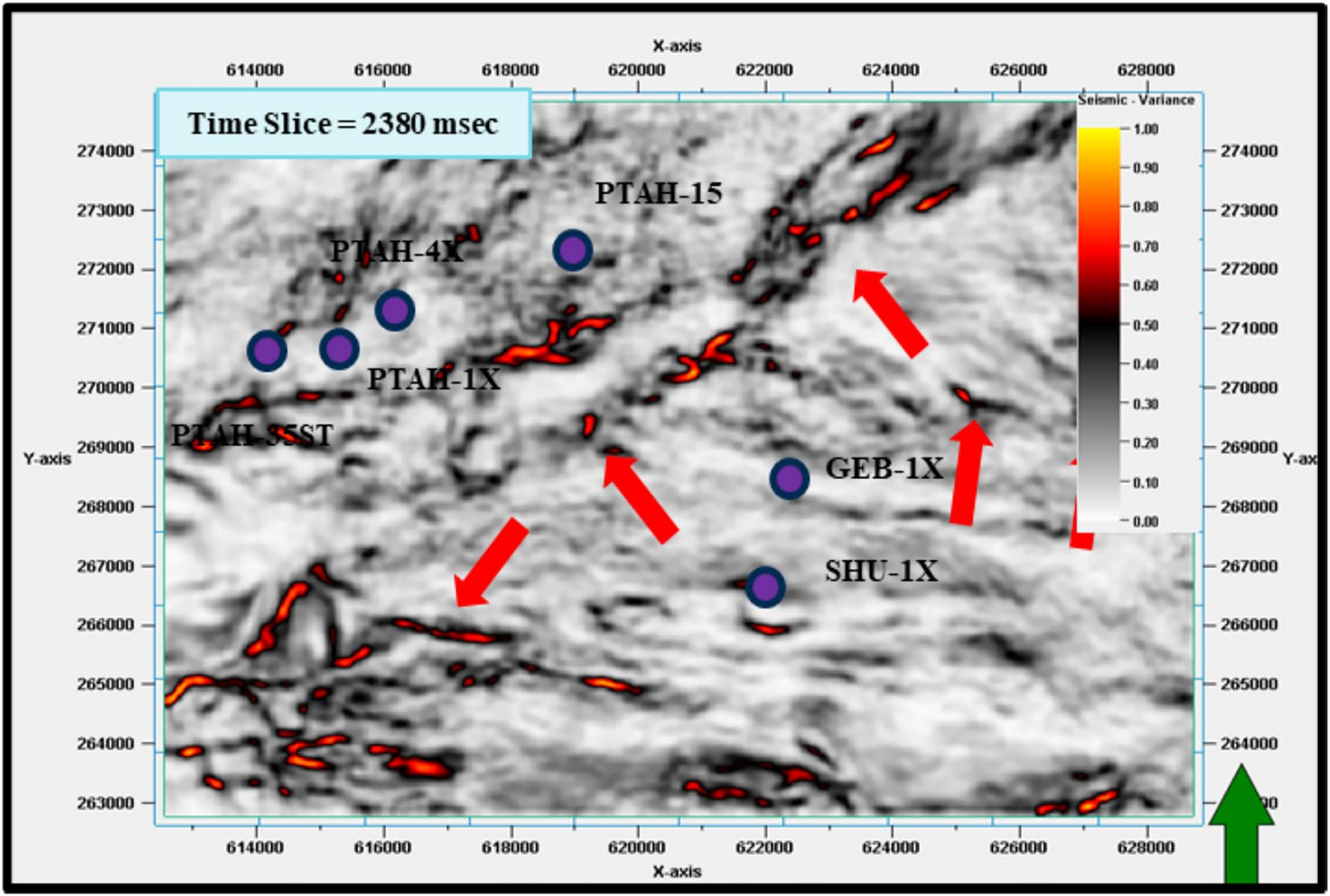
Variance attribute slice at the (2380 msec.) corresponding to Shiffah B SD unit showing structural discontinuities and fault patterns highlighted by red arrows.

The variance attribute was applied to the seismic dataset to enhance the detection of structural discontinuities and fault patterns within the study area. Variance measures the similarity between neighboring seismic traces and is widely used to highlight faults and stratigraphic boundaries^34,35^. Variance slices extracted at the Bahariya, Alam El Bueib-3D and Paleozoic Shiffah B levels clearly reveal a network of linear discontinuities interpreted as fault zones (Figs. 13–15). The attribute highlights subtle faults that are difficult to identify in conventional seismic amplitude sections and confirms the structural framework interpreted from the seismic data. The detected fault trends show a dominant NE–SW orientation with secondary NW–SE trends, consistent with the regional structural pattern of the Northern Western Desert. These discontinuities control reservoir compartmentalization and are consistent with the fault patterns observed on the seismic sections (Figs. 9–12).

#### Structural mapping

**Fig. 16:**
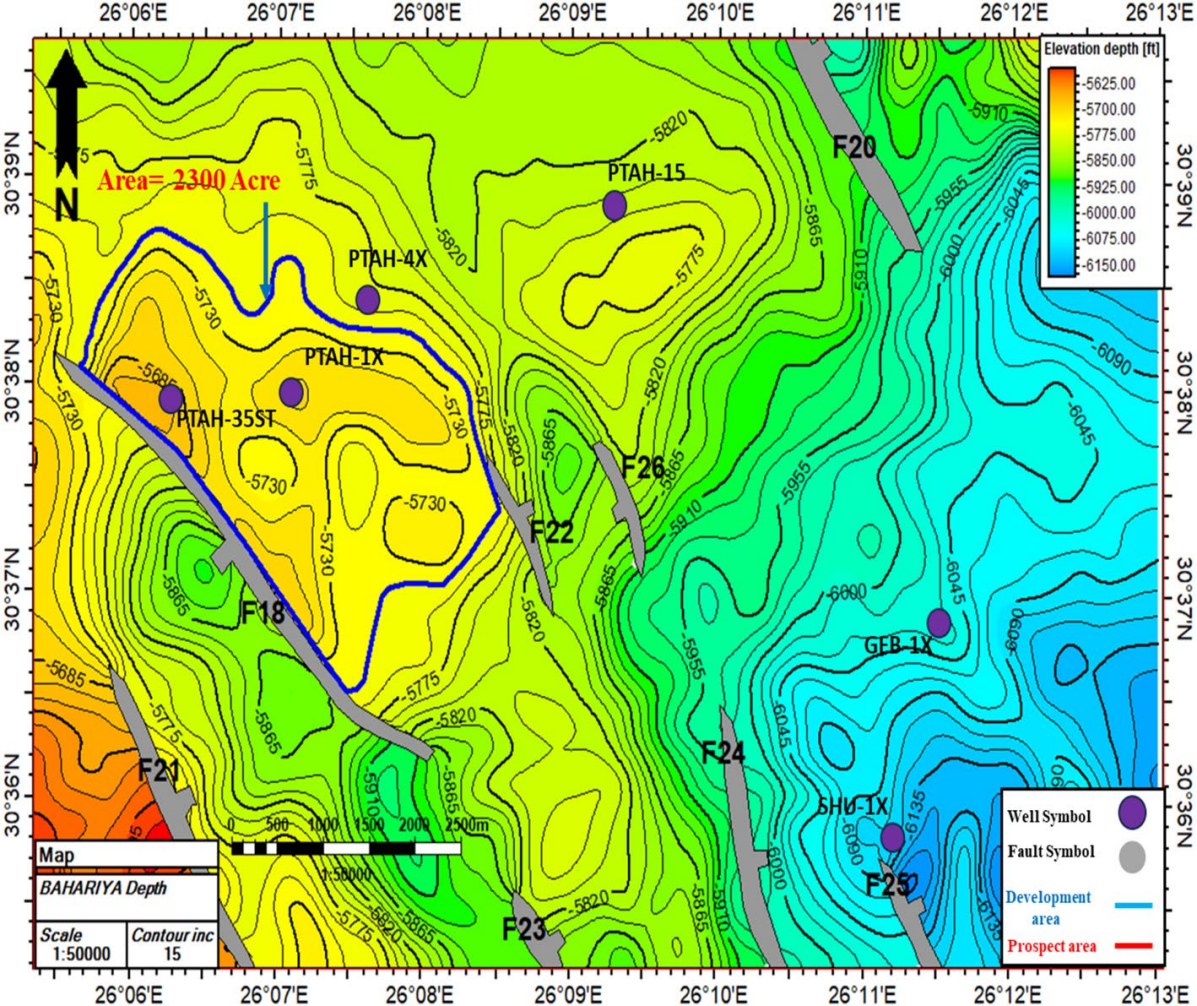
Depth structure contour map for Bahariya Formation.

**Fig. 17:**
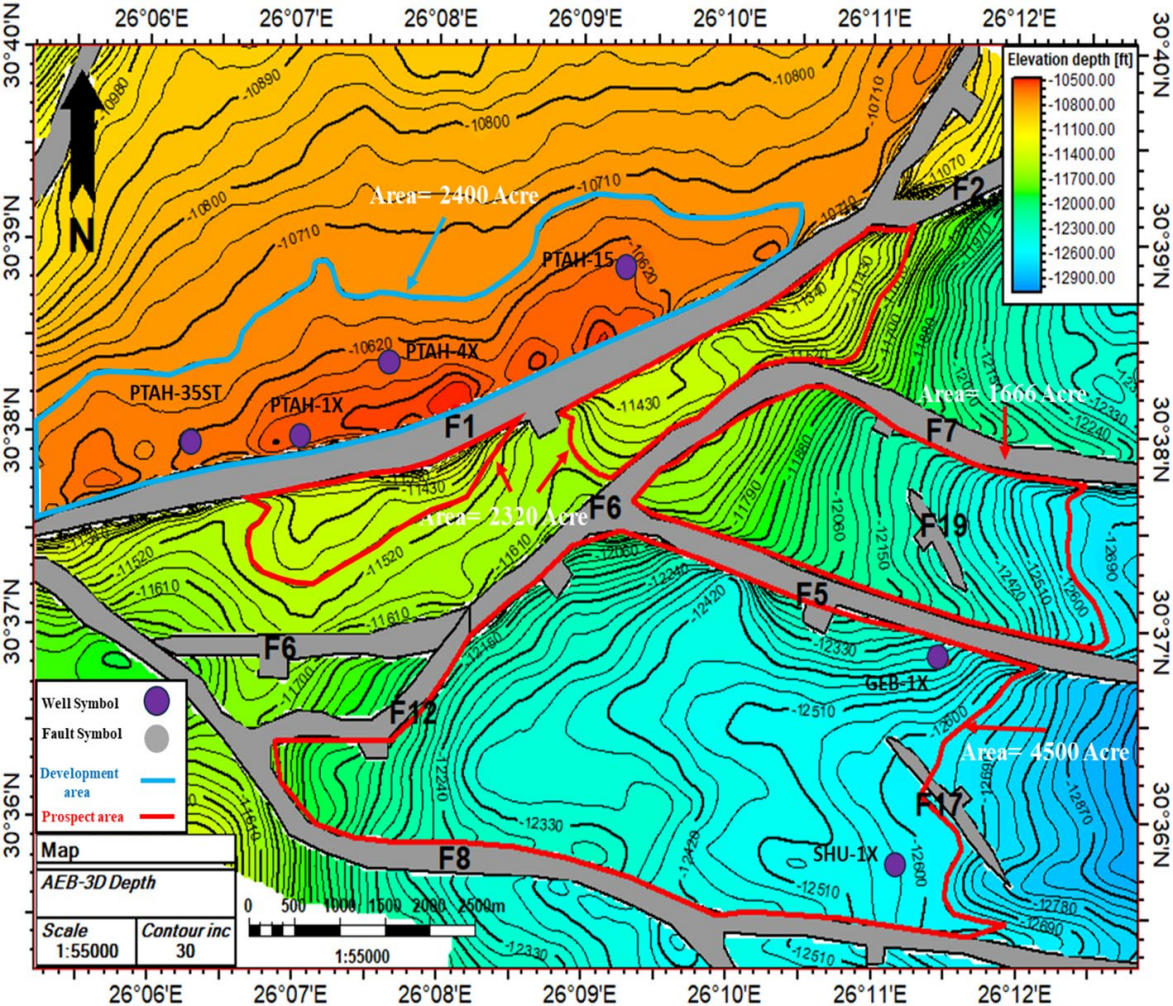
Depth structure contour map for Alam El Bueib-3D unit.

Depth-converted structure maps of the Bahariya, Alam El Bueib-3D, and Paleozoic Shiffah B SD intervals reveal a fault-controlled structural framework expressed by differential block displacement and localized closure development. Structural contour patterns show systematic depth variation governed by normal faulting, consistent with extensional basin architecture. The Bahariya depth structure map (Fig. 16) displays moderate structural relief characterized by stepped fault blocks and gently varying contour gradients. Structural highs correspond to upthrown blocks where contour spacing is relatively broad, indicating low dip angles, while downthrown blocks exhibit tighter contour spacing reflecting steeper gradients. Linear contour discontinuities mark fault traces, producing abrupt depth offsets indicating extensional fault system. Local contour closures occur on elevated blocks, where reflector continuity is preserved across fault-bounded domains. The Bahariya structure map reveals a fault-bounded closure covering approximately 2300 acres. The structural crest occurs at about −5700 ft TVDSS, while the interpreted spill point is located near −5755 ft TVDSS, giving a structural relief of approximately 55 ft. The Alam El Bueib-3D depth structure map (Fig. 17) exhibits more pronounced segmentation, with contour deflection and closure development controlled by major fault orientations. Depth gradients increase near fault intersections, producing elongated structural highs aligned with dominant fault trends. These geometries reflect differential displacement and fault linkage, generating compartmentalized structural domains consistent with layered extensional deformation. The main closure extends over roughly 2400 acres at the upthrown side of Ptah major fault (F1) represents in blue line within the northern part of the study area. The structural crest occurs near the central part of the closure at approximately −10500 ft TVDSS, whereas the interpreted spill point is located along the northeastern margin of the structure near the −10680 ft TVDSS contour, this geometry defines a structural relief of about 180 ft between the crest and spill point. The secondary closures represented by red lines range between approximately 1660, 2320, and 4500 acres, corresponding to discrete fault-bounded compartments and considered favorable targets for future drilling.

**Fig. 18:**
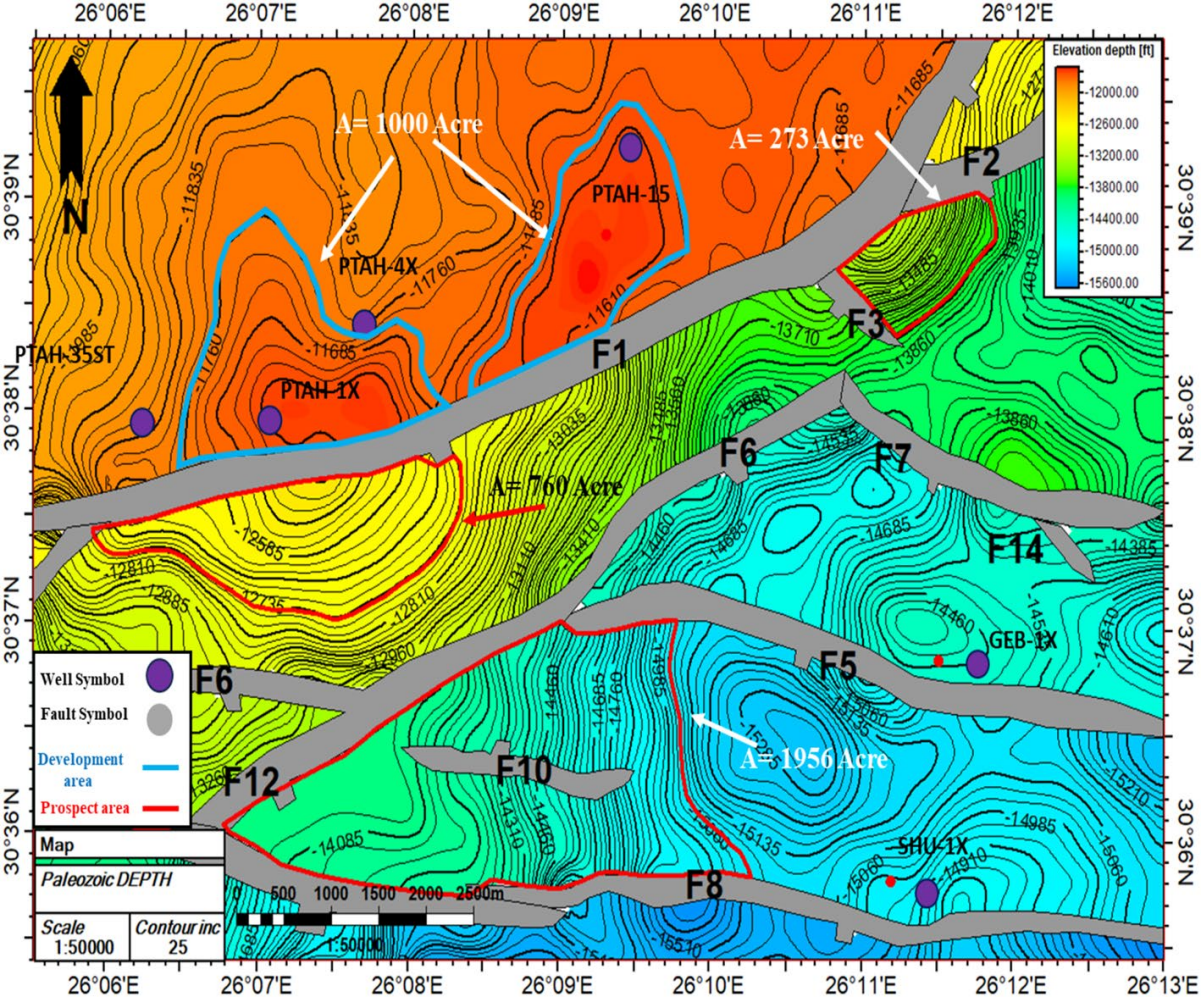
Depth structure contour map for Paleozoic Shiffah B SD unit.

The Paleozoic Shiffah structure map (Fig. 18) shows the greatest depth range and strongest expression of fault-controlled relief. Regional depth gradients are superimposed on block segmentation, where contour convergence outlines closures developed on structurally elevated and adjacent fault-bounded domains. The principal closure has two culminations and occupies approximately 1000 acres; it is represented by blue lines, while secondary closures range between roughly 273, 760 and 1956 acres, represents in red lines. The principle Clouser is developed on a structurally elevated block north of the major (F1) fault, the structural crest occurs near the central part of the two closures at approximately −11600 ft TVDSS, while the interpreted spill points are −11630 and 11760 ft TVDSS. This configuration defines a structural relief of approximately (30-160 ft). The closure geometry suggests a favorable structural trap where hydrocarbons accumulate within the Paleozoic Shiffah B sandstone reservoir. The secondary closures are considered favorable targets for future drilling.

Comparison of the three structural maps indicates consistent spatial alignment of fault-controlled domains across stratigraphic levels. Elevated blocks identified in the Bahariya surface correspond to structurally high areas in the Alam El Bueib-3D and Paleozoic Shiffah B SD intervals, while downthrown domains maintain similar geometric relationships. Variations in contour spacing reflect stratigraphic differences in displacement expression rather than changes in fault orientation. Collectively, the structural maps define a coherent three-dimensional framework characterized by block segmentation, closure development, and depth gradients typical of extensional fault systems. Although detailed quantitative fault throw measurements and displacement statistics were not systematically calculated in this study, interpretation of seismic sections indicates that fault displacement varies along strike and with depth, consistent with segmented normal fault systems.

#### Geological cross-section

**Fig. 19:**
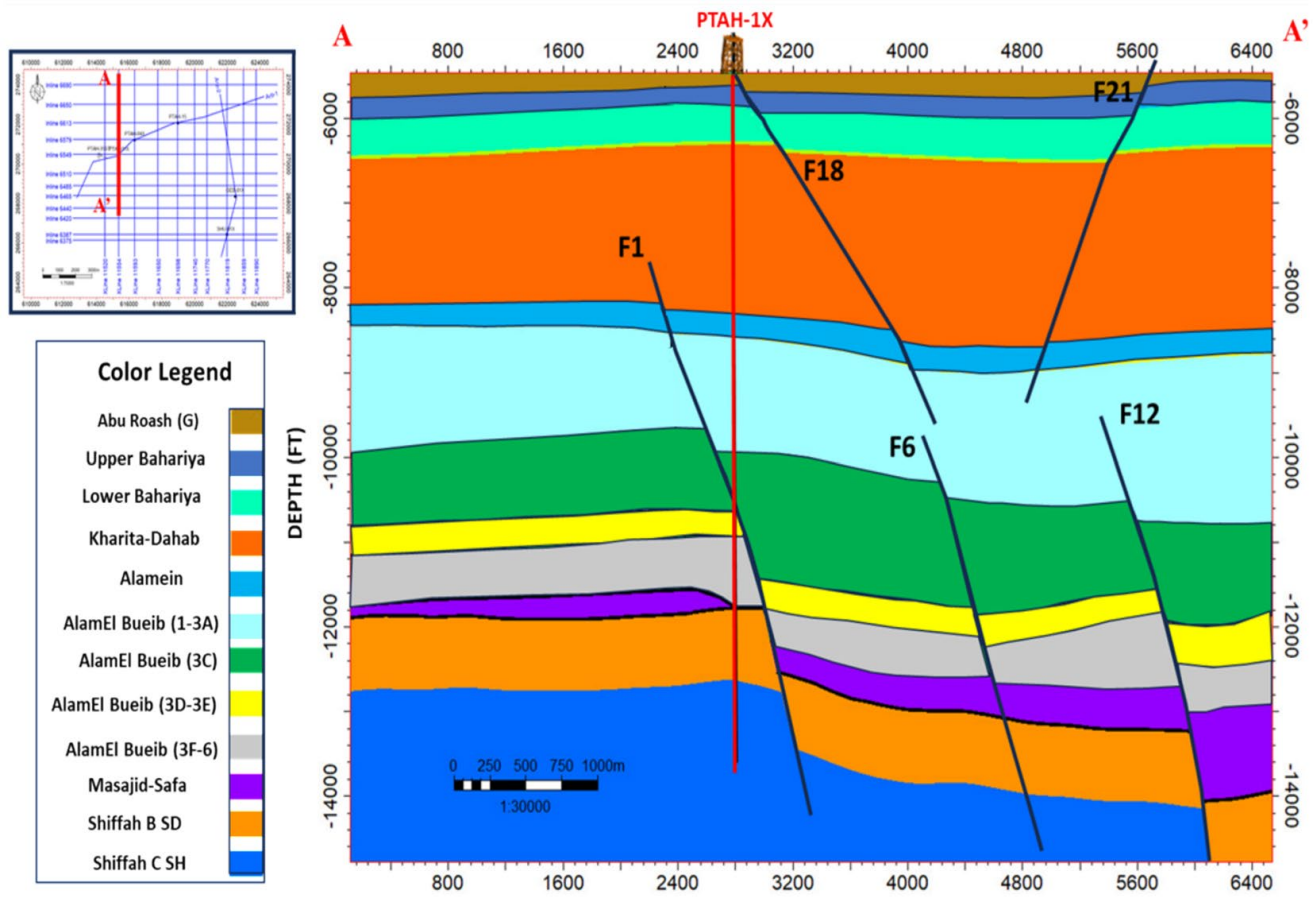
Geological cross-section A–A′ illustrating the structural framework of the Ptah Field and lithological juxtaposition along major fault planes (Vertical exaggeration = 1).

The geological cross-section along line A–A′ (Fig. 19) illustrates the structural framework of the Ptah Field and the vertical relationship between the principal stratigraphic units and the interpreted fault system. The section shows a set of southwest normal faults (F18 and F21) affecting Bahariya formation, while south and southeast-dipping normal faults (F1, F6 and F12) affecting the Alam El Bueib, and Paleozoic intervals, forming fault-bounded structural compartments typical of extensional basin architecture. These faults exhibit significant displacement, with estimated fault throws ranging approximately between 200 and 750 ft., producing clear vertical offsets between stratigraphic horizons and strongly influencing the structural configuration of the reservoir units. Structurally elevated blocks correspond to potential hydrocarbon trapping zones, whereas downthrown blocks display deeper reservoir positioning and increased burial depths. In addition to structural displacement, the cross section reveals noticeable thickness variations across the fault blocks, where several intervals locally thicken toward the downthrown sides of the structures. This pattern reflects syn-tectonic subsidence and depositional accommodation associated with extensional fault activity. Fault displacement also results in lithological juxtaposition between different stratigraphic units. In particular, the Lower Bahariya sandstone reservoir is juxtaposed against the Basel Upper Bahariya shale, Alam El Bueib-3D sandstone reservoir is juxtaposed against the shale-dominated Alam El Bueib -3C interval, while the Paleozoic Shiffah B sandstone reservoir is juxtaposed against shale-rich units of the Alam El Bueib-3F to Alam El Bueib-6 intervals. These shale-dominated units may act as effective lateral sealing elements along the fault planes, enhancing reservoir compartmentalization and contributing to the preservation of hydrocarbons within structurally elevated fault-bounded blocks. Although a quantitative fault seal analysis was not performed in this study, the observed lithological juxtaposition relationships suggest the potential for lateral sealing along the major fault planes. Collectively, the interpreted seismic sections and Geological Cross section define a structurally segmented framework characterized by interacting normal faults, vertical reflector displacement, and stratigraphic juxtaposition, consistent with established models of extensional basin architecture^30,33^.

### Petrophysical results

#### Formation water resistivity and cutoff parameters

**Fig. 20:**
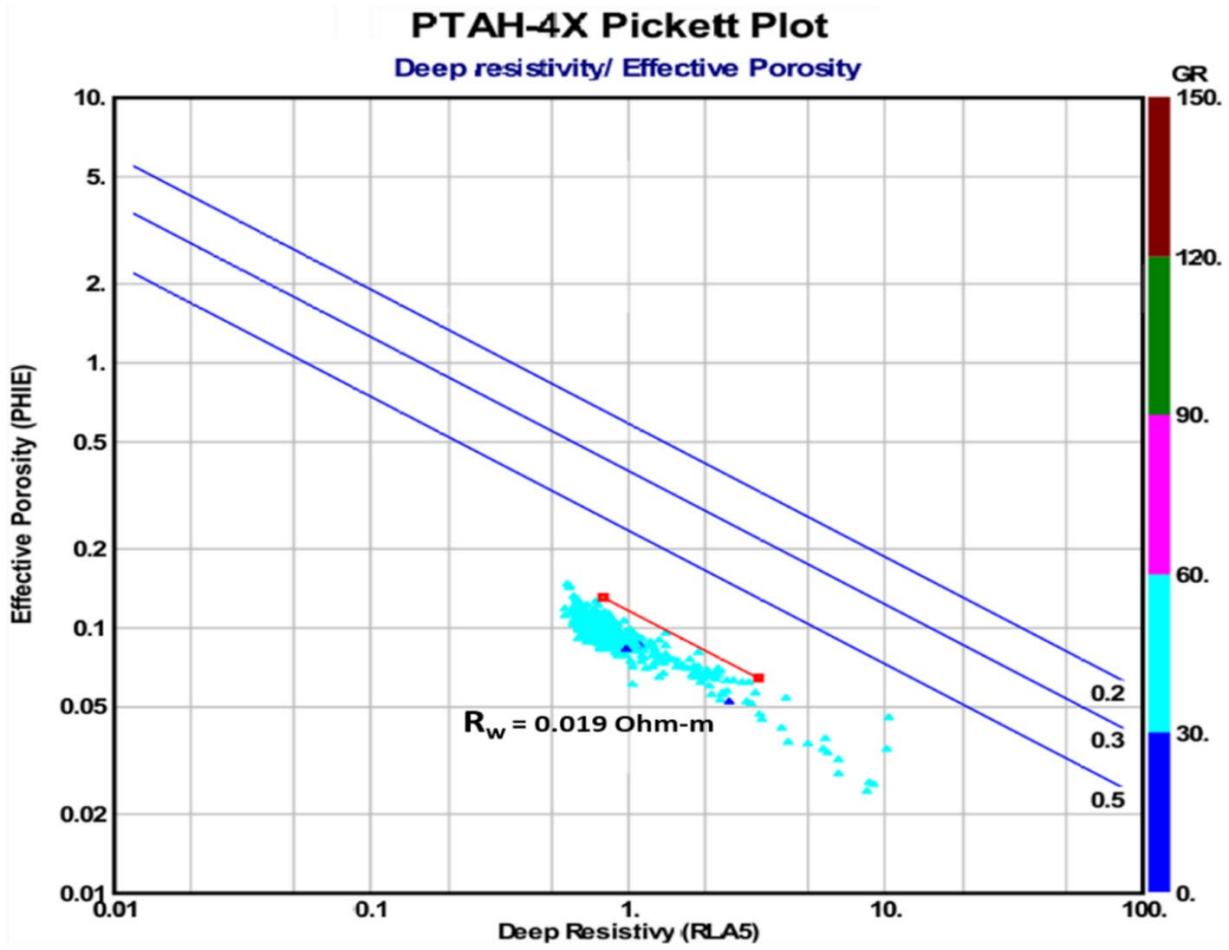
Picket plot for PTAH-4X well showing the value of Rw.

Laboratory-derived formation water resistivity is consistent with Pickett plot^29^ calibration results (Fig. 20), confirming internal consistency of petrophysical evaluation. Standard cutoff criteria were applied across reservoirs to define productive intervals (Table 2).

**Table 2:** The net pay cut off used in the study area.

**Reservoir**	**V**_**sh**_ (%)	**S**_**w**_(%)	**Ф**_**eff**_ (%)
**Bahariya**	<50	<65	>12
**Alam El Beuib**	<35	<50	>8
**Paleozoic**	<35	<35	>6

#### Lithological characterization

**Fig. 21:**
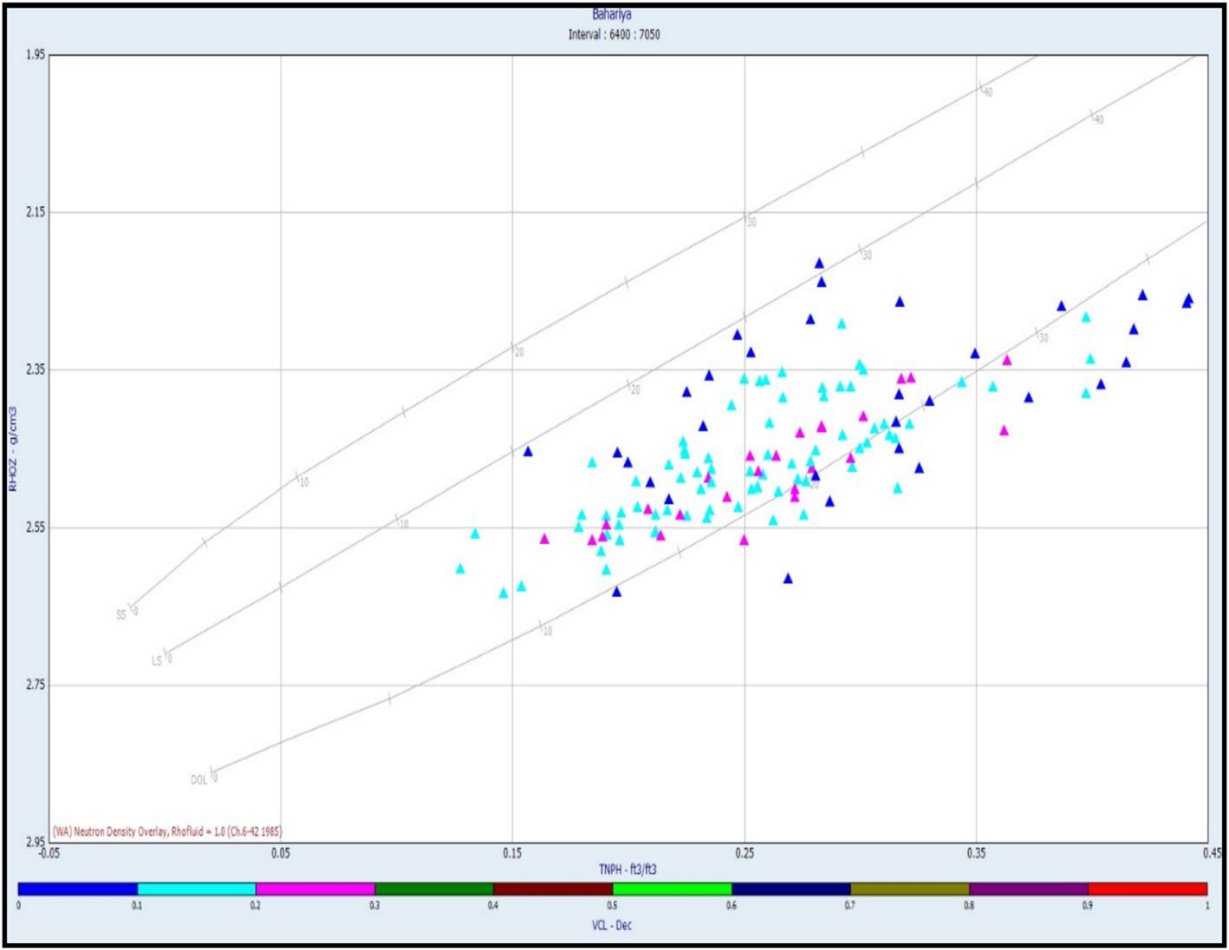
Neutron- density cross plots for Bahariya reservoir in the studied wells.

Neutron–density crossplots for the Bahariya reservoir (Fig. 21) indicate that the majority of plotted data points are clustered close to the limestone and dolomite lines, while a limited number of points are distributed between the sandstone and limestone trends. This distribution suggests the presence of mixed lithologies within the reservoir interval. Effective porosity values range from approximately 13% to 22%, indicating moderate to good reservoir quality. The shale volume generally varies between about 5% and 20%, with a few data points showing shale content below 9%. The dispersion of points toward the dolomite trend is interpreted to result from the influence of shale and siltstone interbeds within the formation. In addition, this distribution may reflect the presence of calcareous cement, which causes some data points to shift toward the limestone trend on the crossplot.

**Fig. 22:**
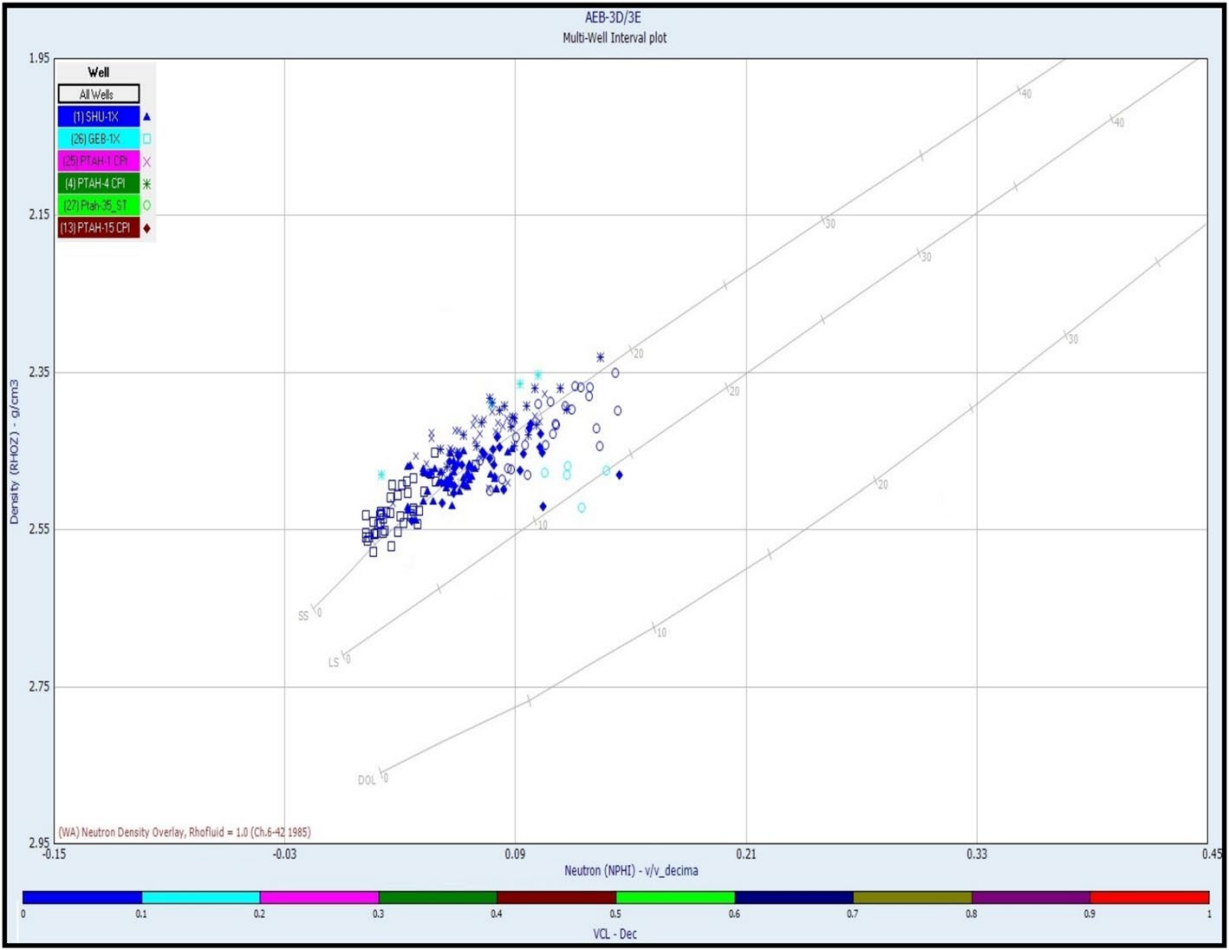
Neutron- density cross plots for Alam El Bueib (AEB-3D/3E) reservoir in the studied wells.

Neutron–density crossplots for the AEB-3D/3E reservoir (Fig. 22) indicate sandstone-dominated lithology with variable shale influence. Most data cluster along sandstone reference trends, while systematic dispersion toward shale-rich zones reflects lithological heterogeneity. Effective porosity values typically range between 5% and 18%.

**Fig. 23:**
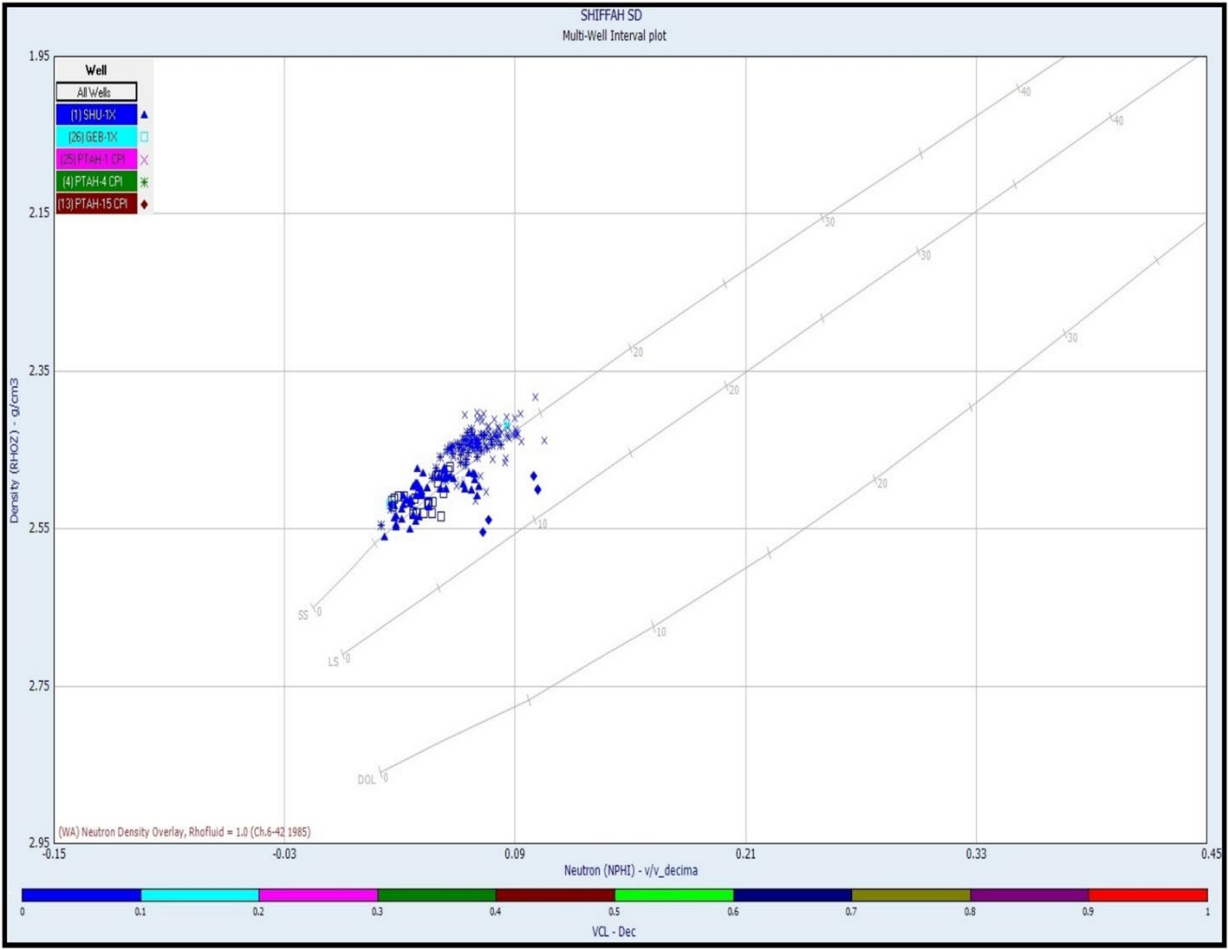
Neutron- density cross plots for Paleozoic Shiffah B SD reservoir in the studied wells.

Crossplots for the Paleozoic Shiffah B reservoir (Fig. 23) similarly shows sandstone-dominated intervals with moderate dispersion attributable to shale interbeds. Porosity ranges from approximately 5% to 17%, indicating variability in reservoir quality among wells.

#### Vertical petrophysical trends

**Fig. 24:**
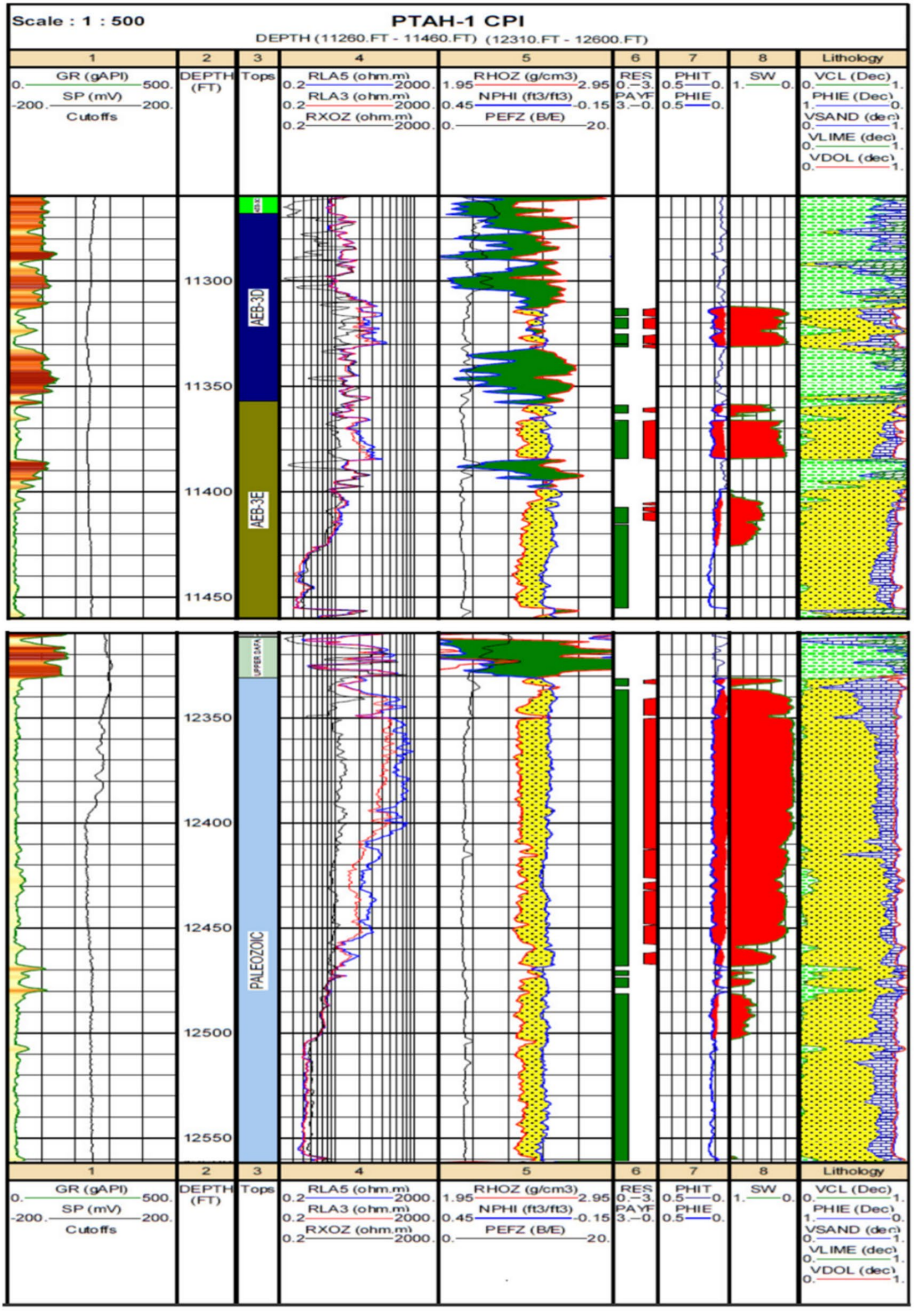
Litho saturation cross plot PTAH- 01X well.

**Fig. 25:**
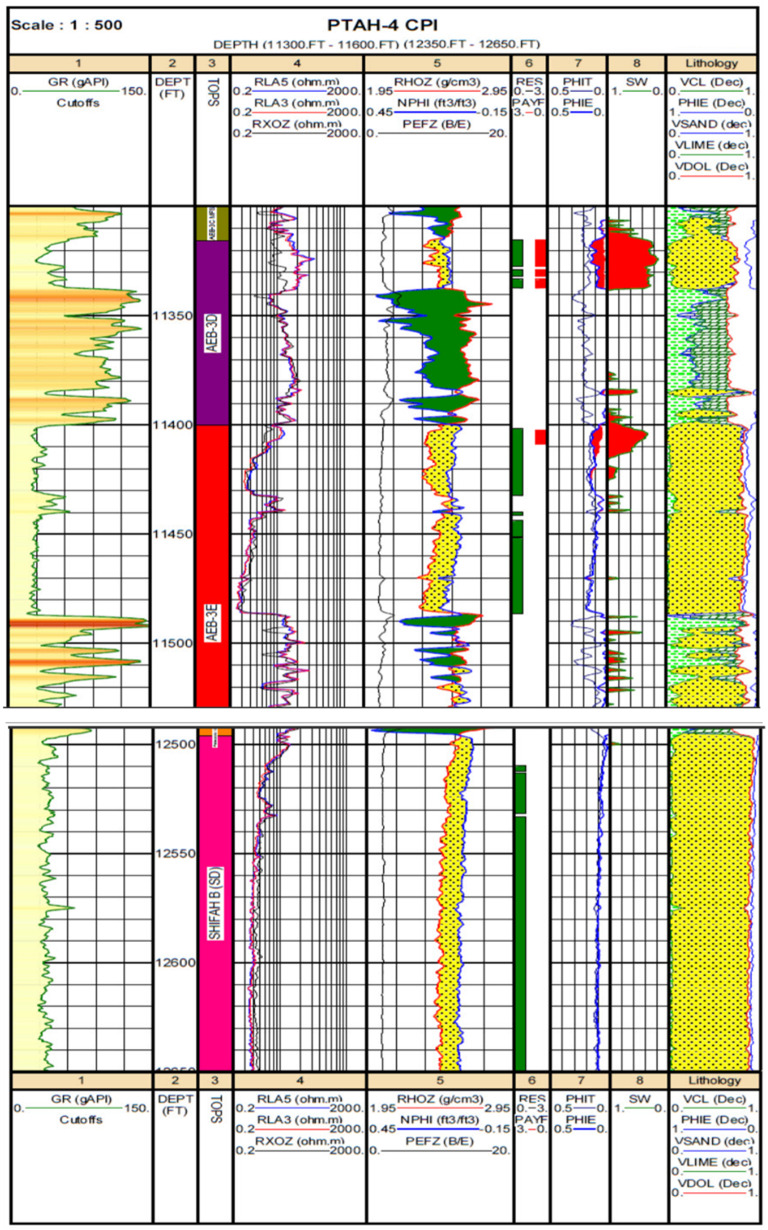
Litho saturation cross plot PTAH- 04 well.

**Fig. 26:**
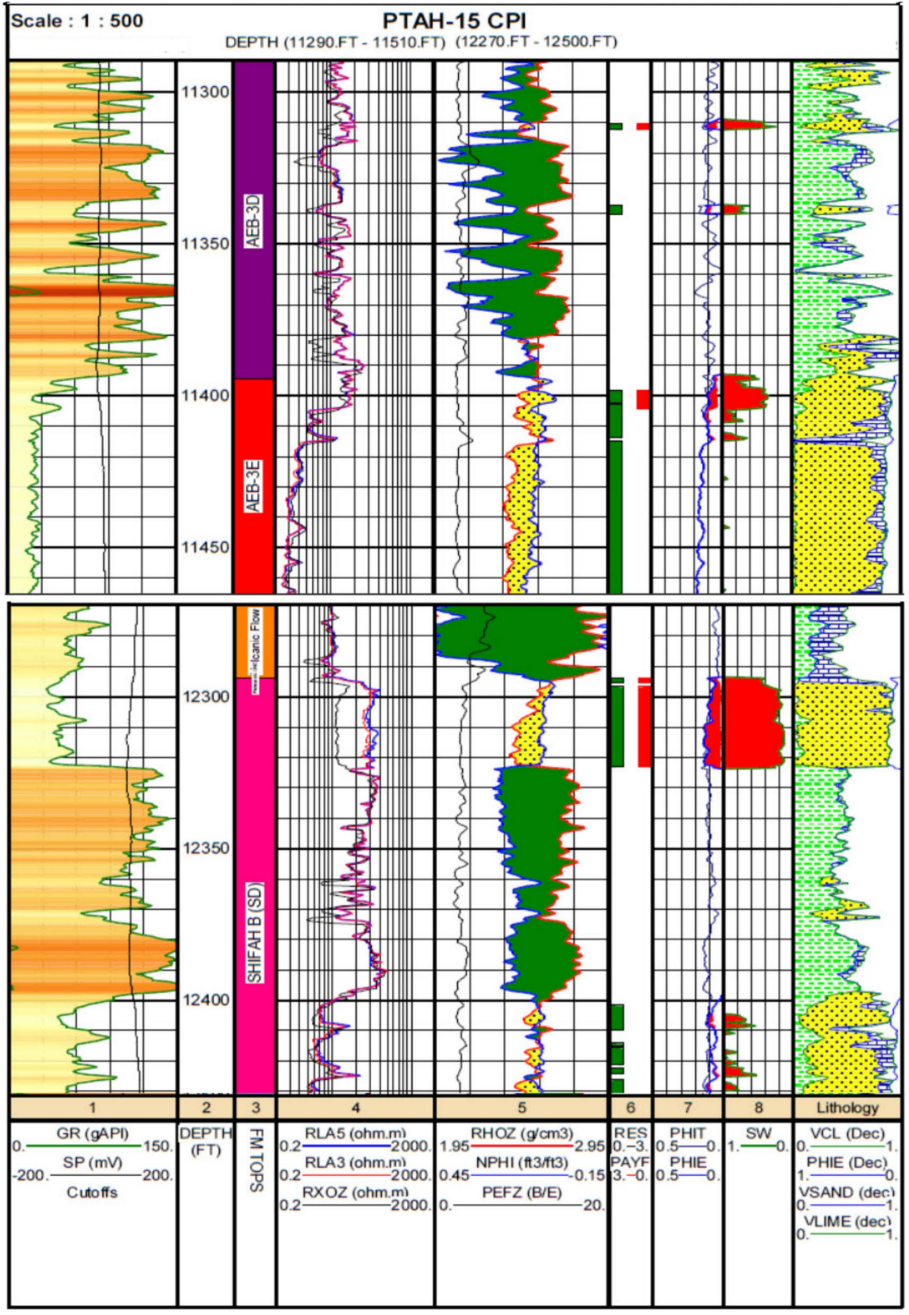
Litho saturation cross plot PTAH- 15 well.

**Fig. 27:**
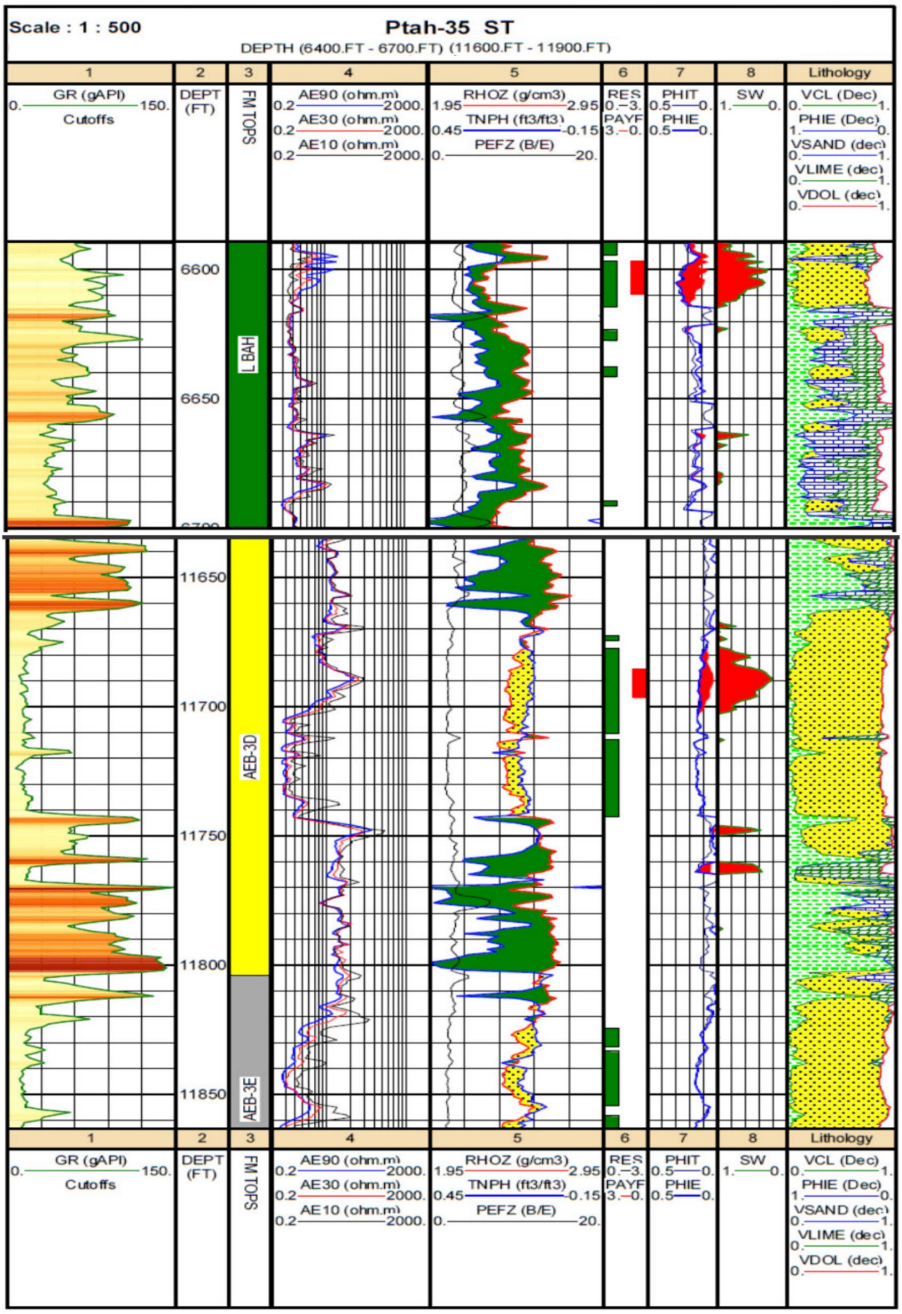
Litho saturation cross plot PTAH- 35ST well.

**Fig. 28:**
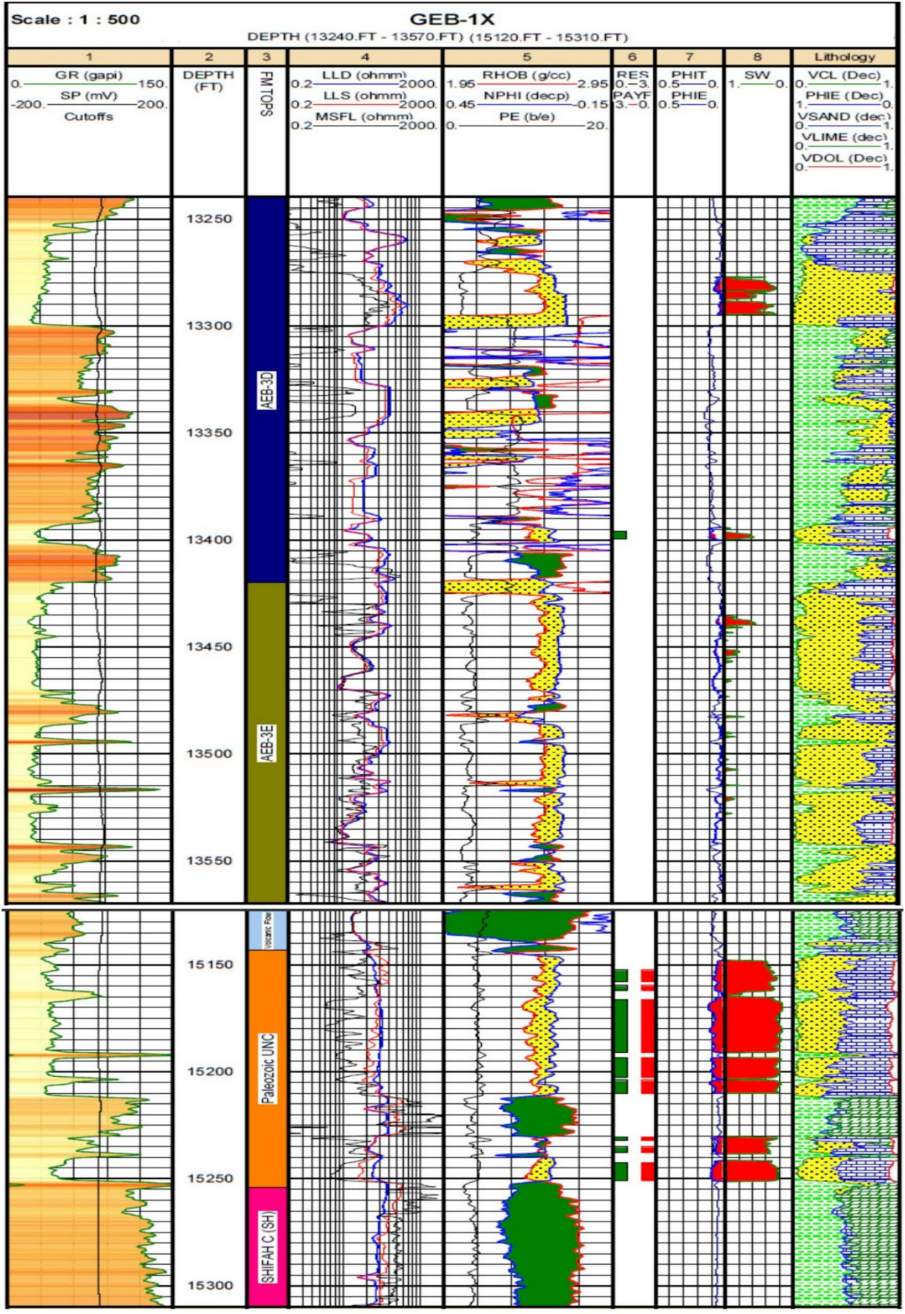
Litho saturation cross plot GEB- 01X well.

**Fig. 29:**
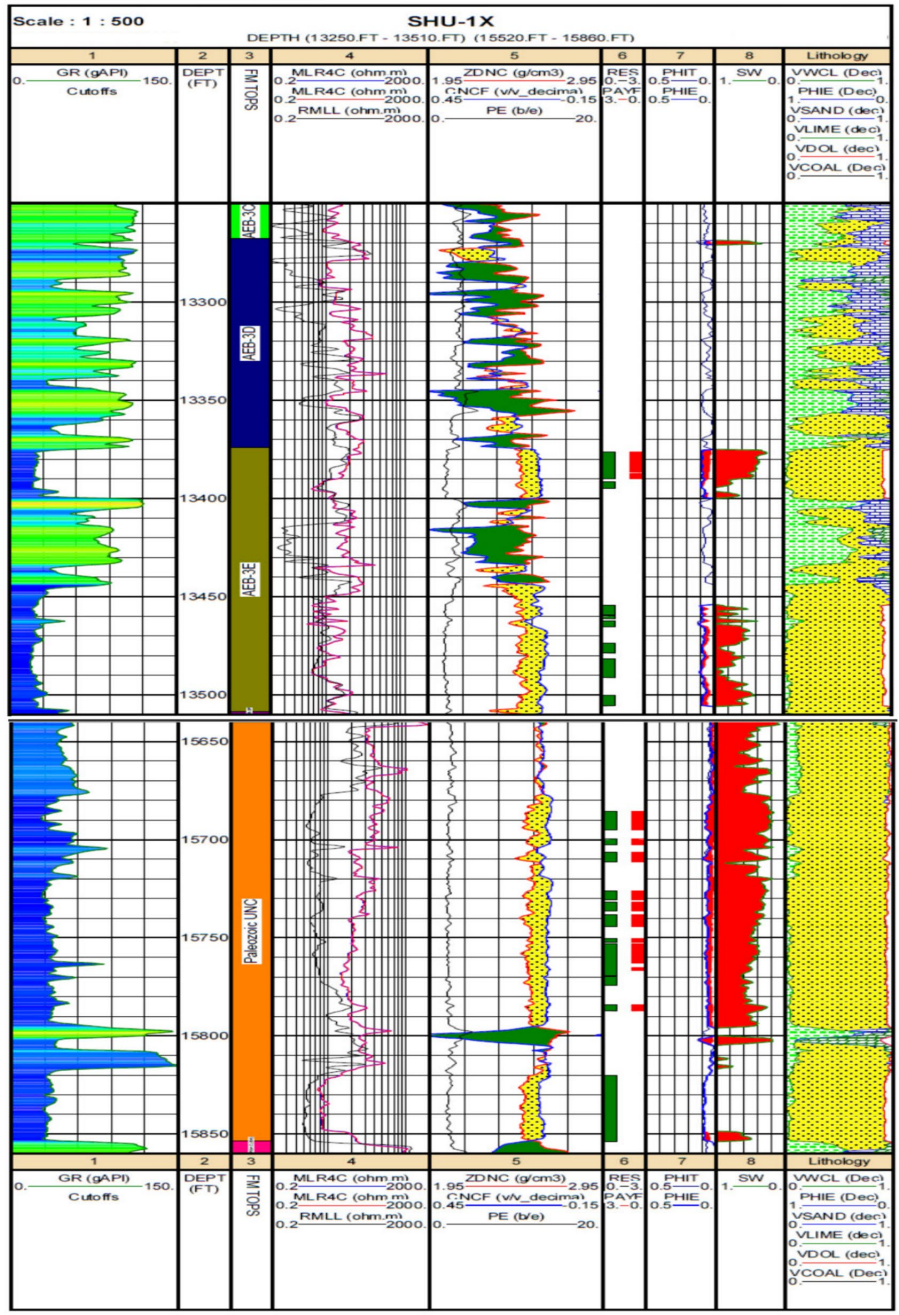
Litho saturation cross plot SHU-01X well.

Litho-saturation plots (Figs. 24–29) demonstrate consistent vertical relationships among shale volume, effective porosity, and water saturation. Increased shale content corresponds to reduced porosity and elevated water saturation within Bahariya, AEB-3D/3E and Paleozoic Shiffah B SD intervals. These plots reveal alternating sandstone–shale successions producing vertical variability in reservoir properties. Summary statistics for these relationships are presented in (Tables 3, 4 and 5).

**Table 3:** The petrophysical analysis for Bahariya reservoir.

**Well**	**Reservoir**	**Thickness (FT)**	**Net Pay** **(FT)**	**Net/ Gross** **(%)**	**V**_**sh**_ **(%)**	**PHIE** **(%)**	**S**_**w**_ **(%)**	**S**_**h**_ **(%)**
**PTAH-35ST**	**Bahariya**	**675**	**13**	**1.92**	**9.1**	**23.2**	**41.7**	**58.3**

**Table 4:** The petrophysical analysis for Alam El Bueib (AEB-3D/3E) reservoir.

**Well**	**Reservoir**	**Thickness (FT)**	**Net Pay** **(FT)**	**Net/ Gross** **(%)**	**V**_**sh**_ **(%)**	**PHIE** **(%)**	**S**_**w**_ **(%)**	**S**_**h**_ **(%)**
**PTAH-01X**	**Alam El Bueib** **(AEB-3D/3E) Unit**	**170**	**38**	**22**	**3.5**	**10.8**	**24.8**	**75.2**
**PTAH-04X**		**265.4**	**28**	**11**	**4.7**	**10.8**	**24.7**	**75.3**
**PTAH-15**		**216.37**	**7.5**	**3.5**	**8.34**	**9.75**	**37.73**	**62.27**
**PTAH-35ST**		**436.2**	**19**	**4.4**	**5.05**	**12.12**	**31.96**	**68.04**
**SHU-01X**		**240.8**	**13**	**5.4**	**3.9**	**8.5**	**38.32**	**61.68**
**GEB-01X**		**482.62**	**0**	**0**	**50**	**7.8**	**100**	**0**

**Table 5:** The petrophysical analysis for Paleozoic (Shiffah B SD) reservoir.

**Well**	**Reservoir**	**Thickness (FT)**	**Net Pay** **(FT)**	**Net/ Gross** **(%)**	**V**_**sh**_ **(%)**	**PHIE** **(%)**	**S**_**w**_ **(%)**	**S**_**h**_ **(%)**
**PTAH-01X**	**Shiffah B** **(Shiffah B SD) Unit**	**694.29**	**129**	**18.58**	**3.8**	**10.6**	**15.8**	**84.2**
**PTAH-04X**		**457.22**	**0**	**0**	**13**	**10.13**	**48**	**52**
**PTAH-15**		**168.12**	**28**	**16.63**	**4.99**	**10.61**	**19.2**	**80.8**
**PTAH-35ST**		**-**	**-**	**-**	**--**	**-**	**-**	**-**
**SHU-01X**		**181.01**	**48.5**	**26.7**	**2.4**	**7**	**33**	**67**
**GEB-01X**		**111.3**	**59.5**	**53.125**	**5.4**	**8.4**	**22.68**	**77.32**

#### Lateral petrophysical distribution

**Fig. 30:**
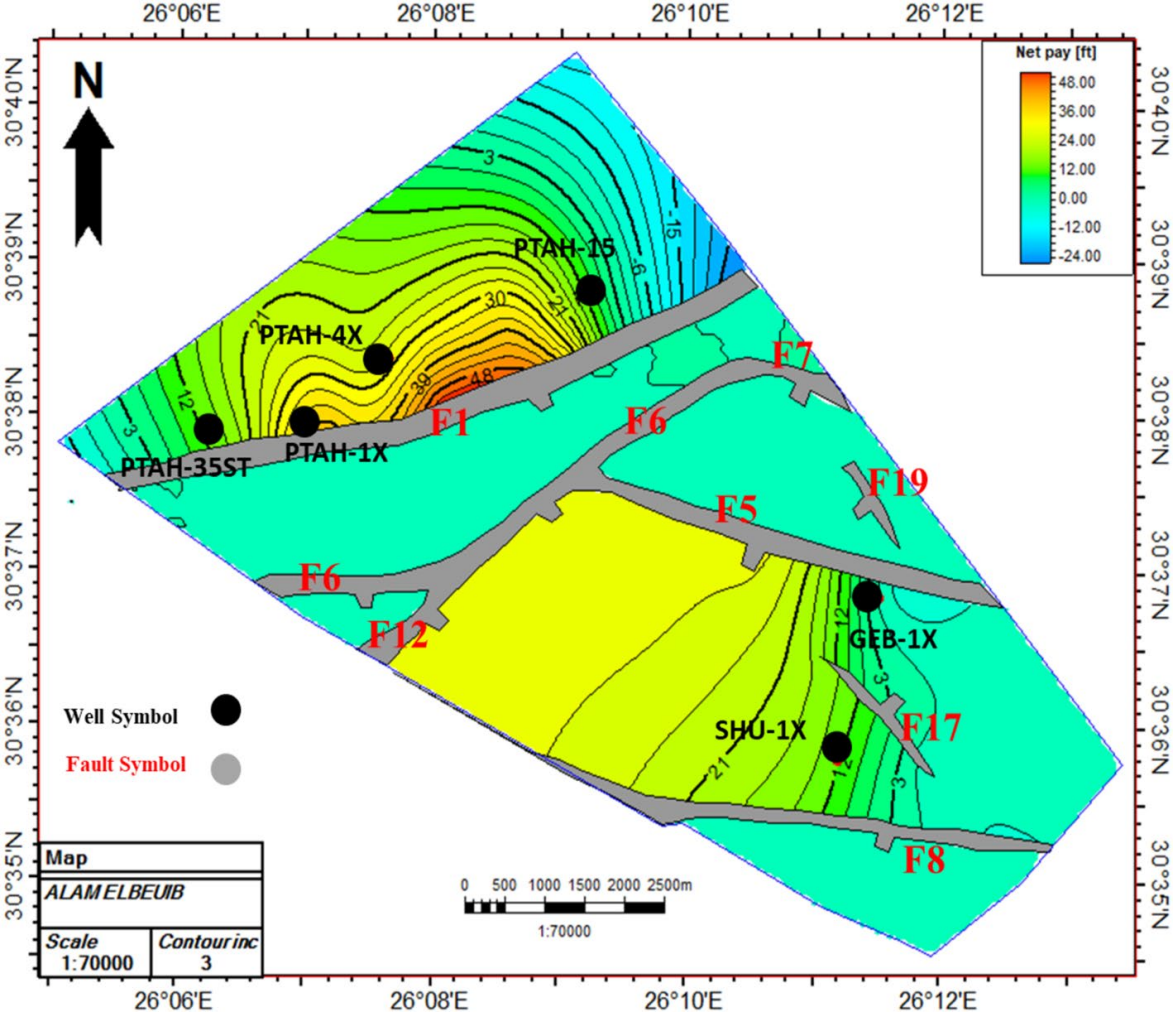
Net Pay variation map for Alam El Bueib (AEB-3D/3E) reservoir.

**Fig. 31:**
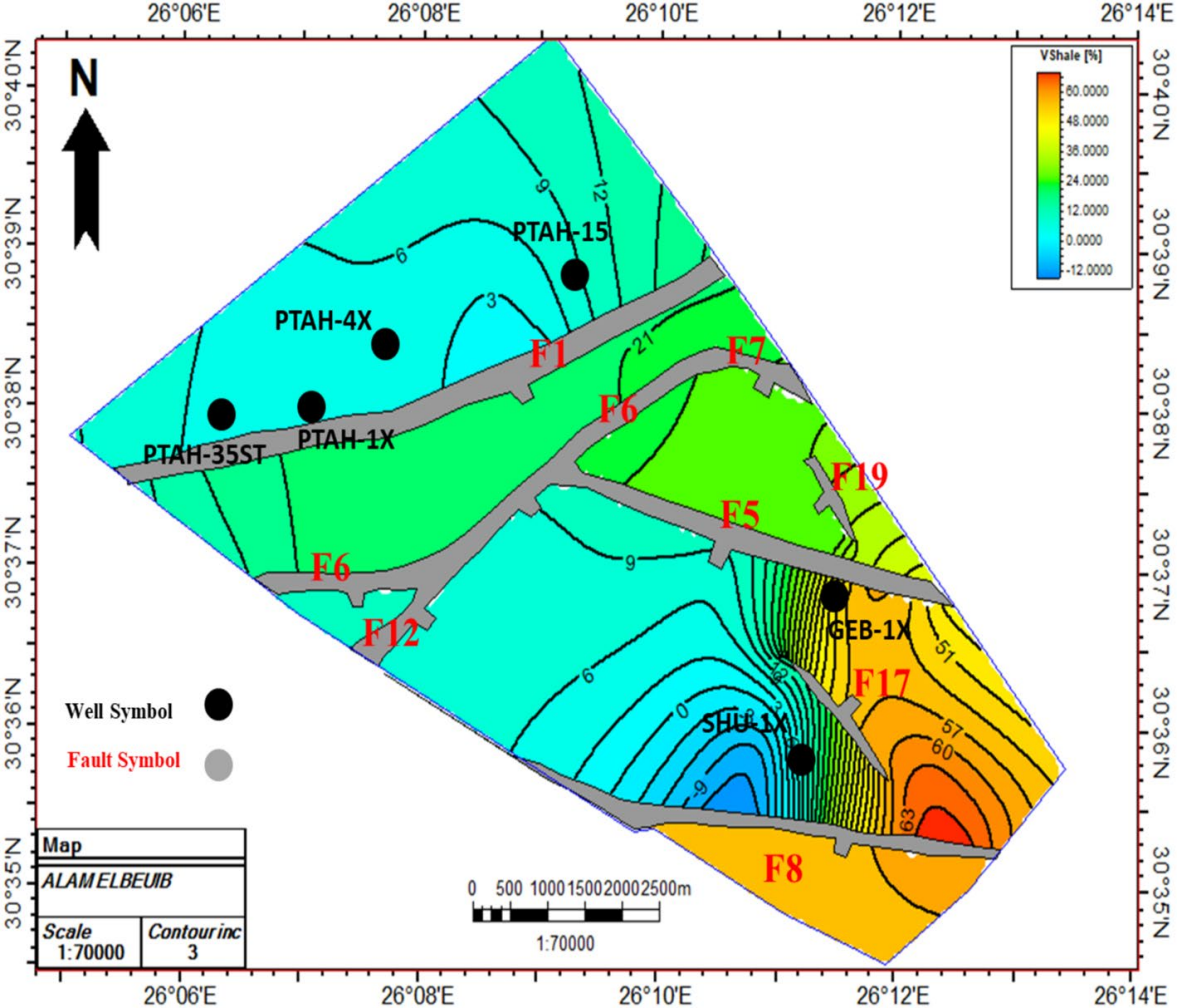
Shale volume variation map for Alam El Bueib (AEB-3D/3E) reservoir.

**Fig. 32:**
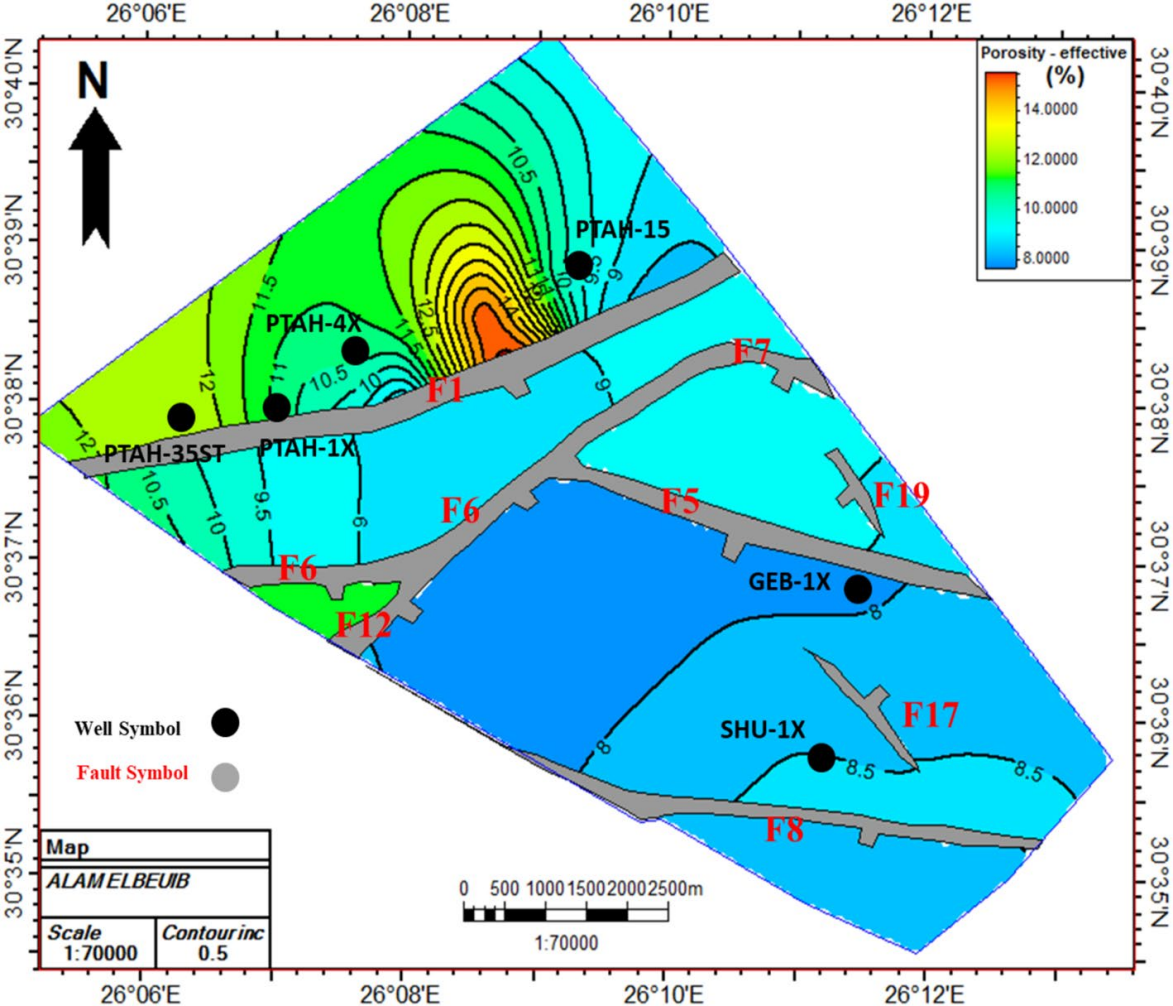
Effective porosity variation map for Alam El Bueib (AEB-3D/3E) reservoir.

**Fig. 33:**
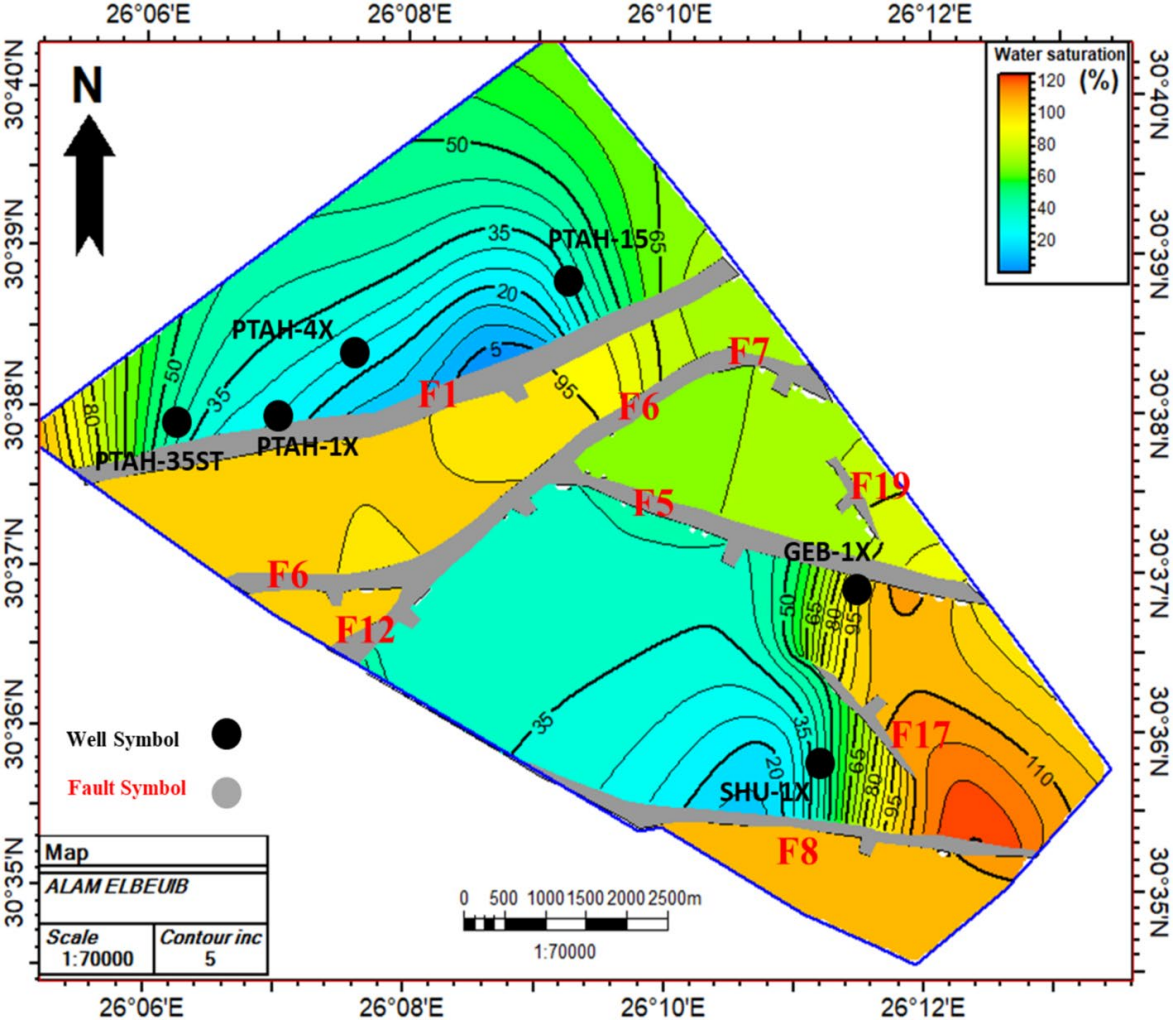
Water saturation variation map for Alam El Bueib (AEB-3D/3E) reservoir.

**Fig. 34:**
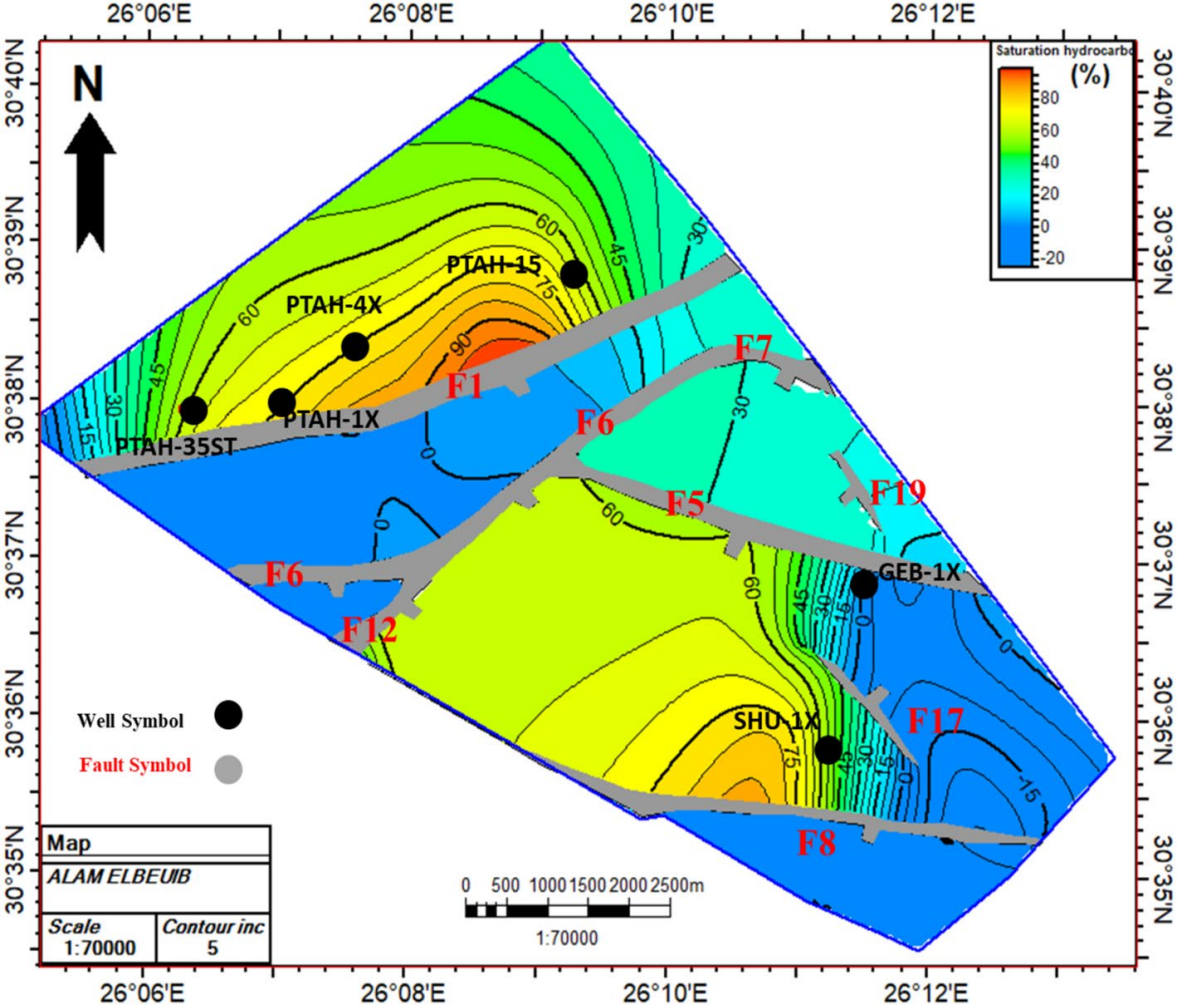
Hydrocarbon saturation variation map for Alam El Bueib (AEB-3D/3E) reservoir.

**Fig. 35:**
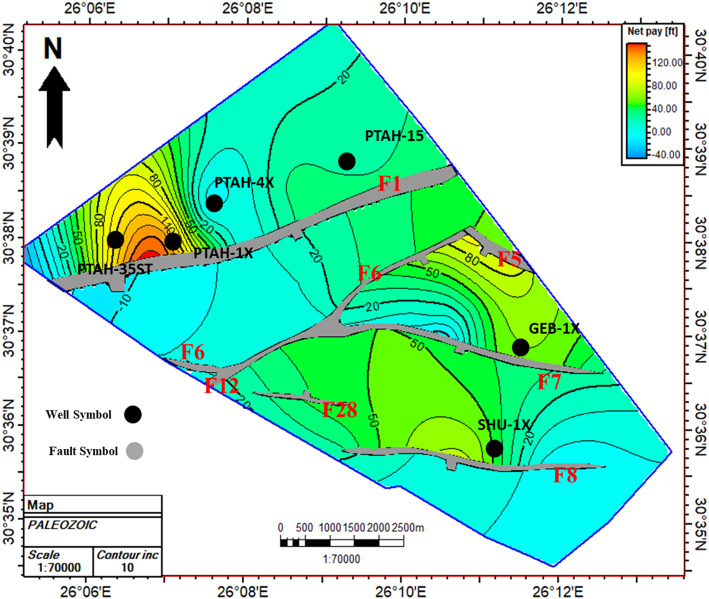
Net Pay variation maps for Paleozoic Shiffah B SD reservoir.

**Fig. 36:**
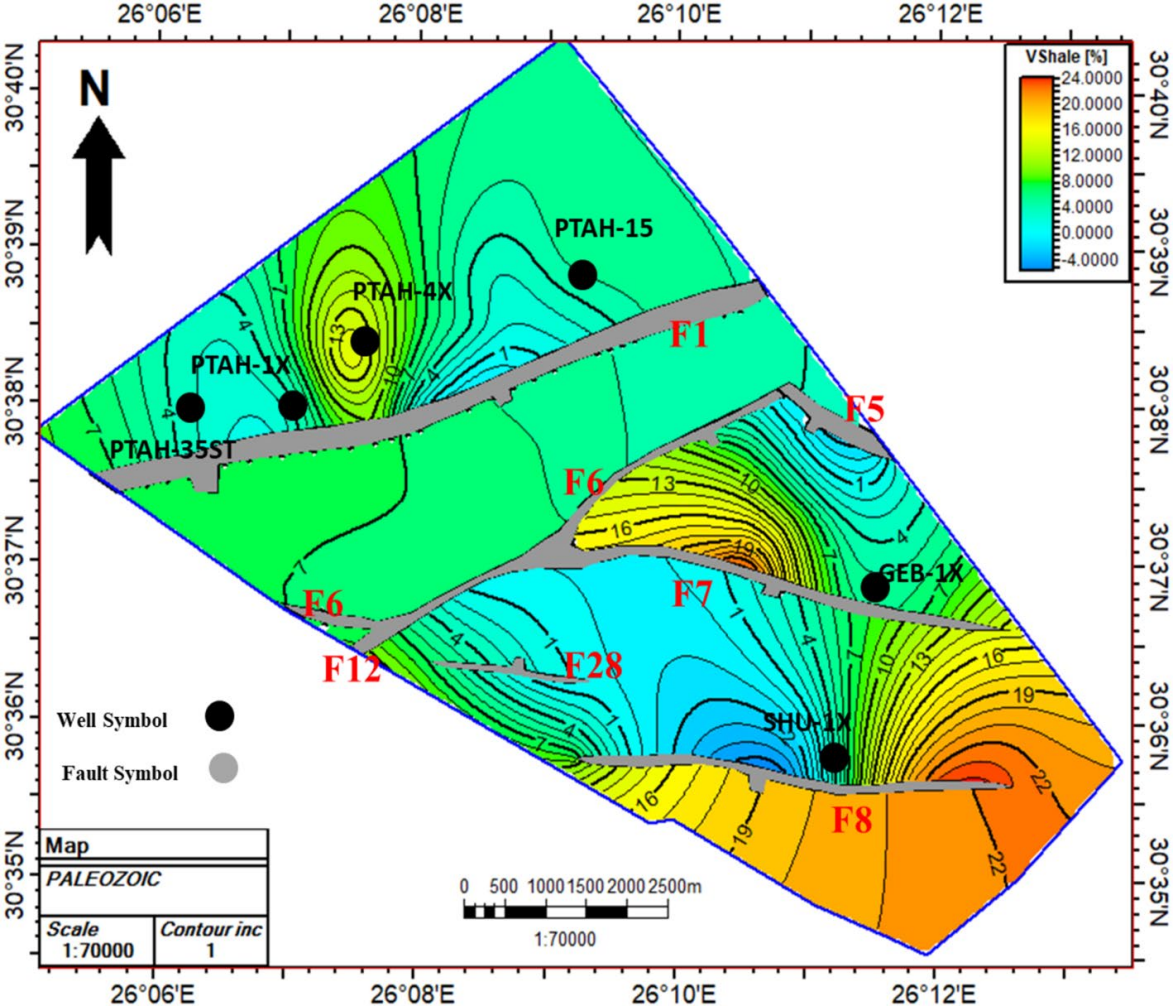
Shale volume variation maps for Paleozoic Shiffah B SD reservoir.

**Fig. 37:**
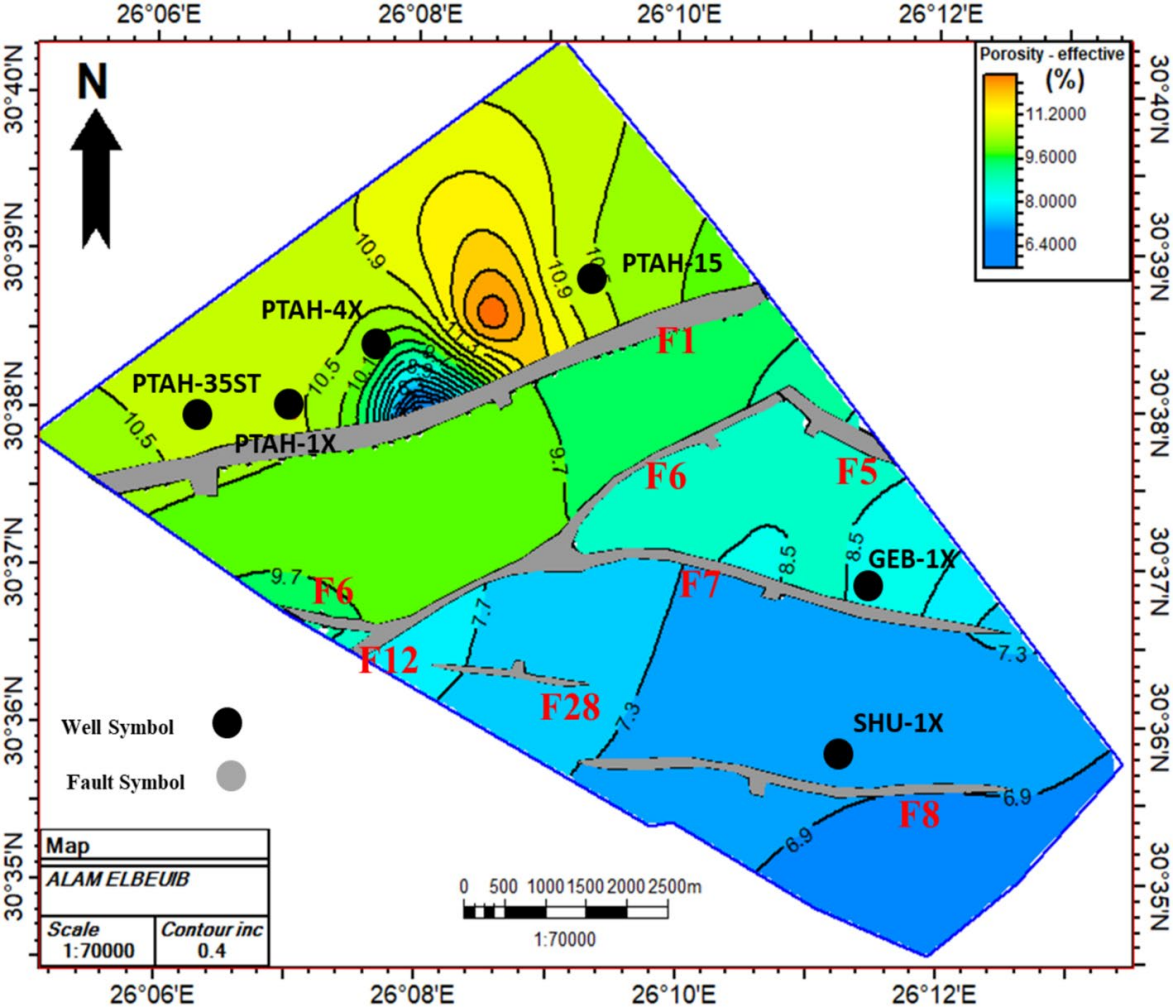
Effective porosity variation maps for Paleozoic Shiffah B SD reservoir.

**Fig. 38:**
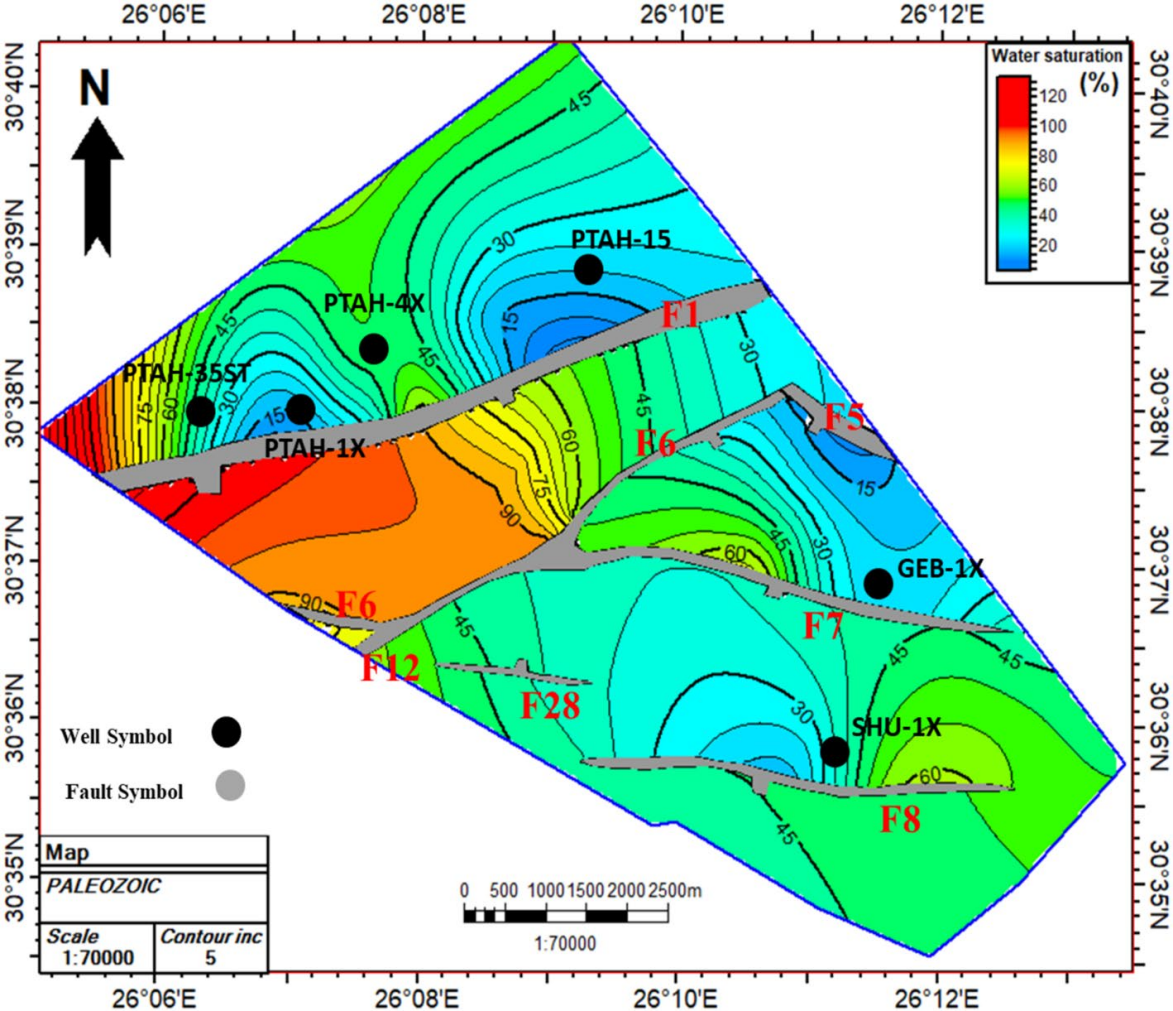
Water saturation variation maps for Paleozoic Shiffah B SD reservoir.

**Fig. 39:**
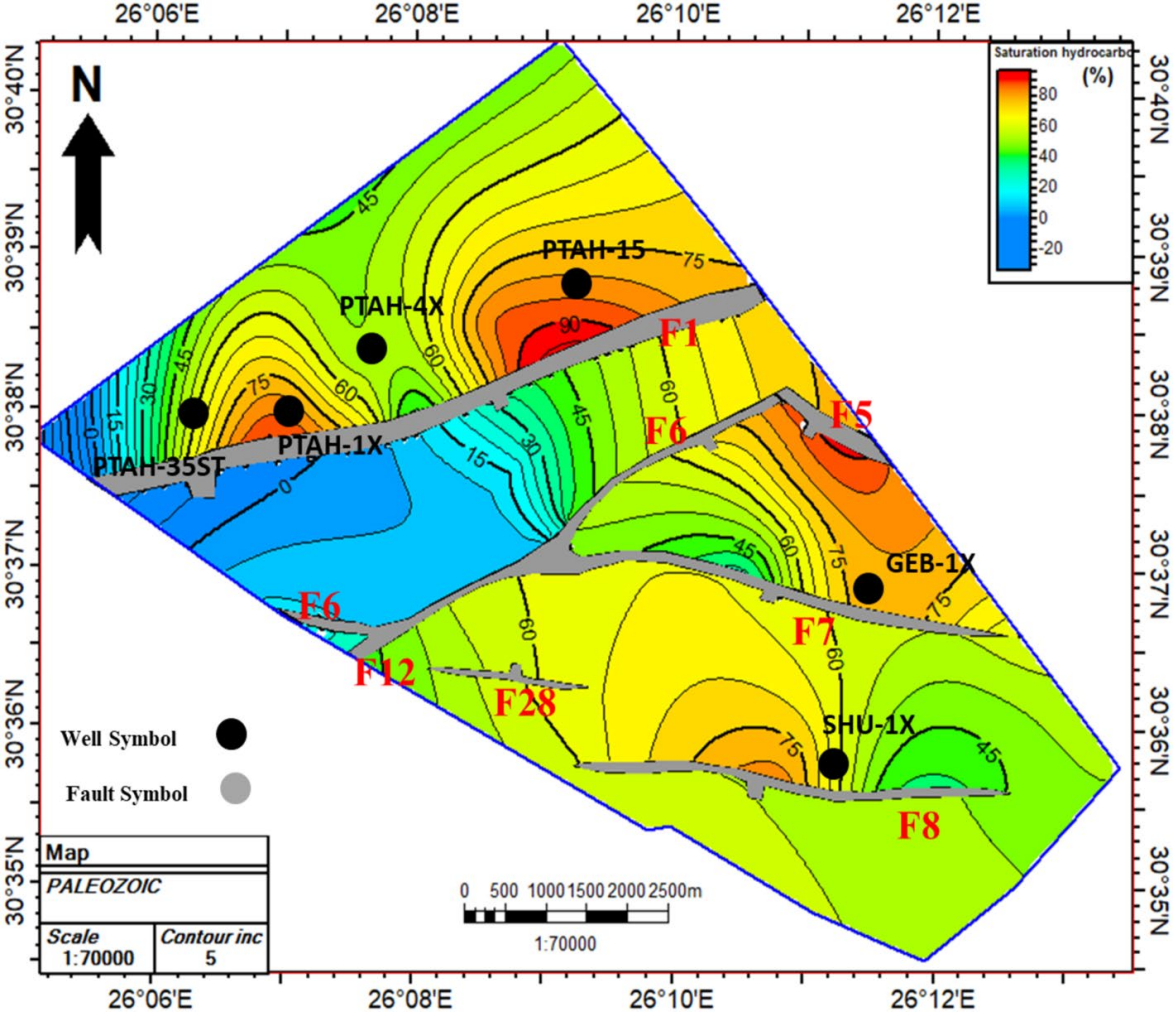
Hydrocarbon saturation variation maps for Paleozoic Shiffah B SD reservoir.

Iso-parametric maps illustrate spatial variability in reservoir properties (Figs. 30–39). In the AEB-3D/3E reservoir, net pay thickness (Fig. 30) increases toward the northwestern sector, coinciding with lower shale content (Fig. 31) and higher effective porosity (Fig. 32). Water saturation (Fig. 33) decreases in these areas, while hydrocarbon saturation (Fig. 34) increases correspondingly. The Paleozoic Shiffah B SD reservoir shows comparable lateral patterns. Net pay thickness (Fig. 35) is greatest in the northwestern region, while shale volume increases toward the south and southeast (Fig. 36). Porosity distribution (Fig. 37) mirrors this trend, with decreasing values in shale-rich areas. Water saturation (Fig. 38) increases where porosity declines, and hydrocarbon saturation (Fig. 39) is concentrated in structurally elevated zones. Although interpolation uncertainty exists near faults and sparsely drilled areas, spatial relationships among mapped parameters remain consistent.

Although the Bahariya Formation may be considered a potential reservoir in the Ptah Field, hydrocarbon occurrence in the available dataset is limited to a single well. Therefore, performing a reliable lateral petrophysical evaluation or constructing iso-parametric maps would not be representative.

### Petrographic and core results

**Fig. 40:**
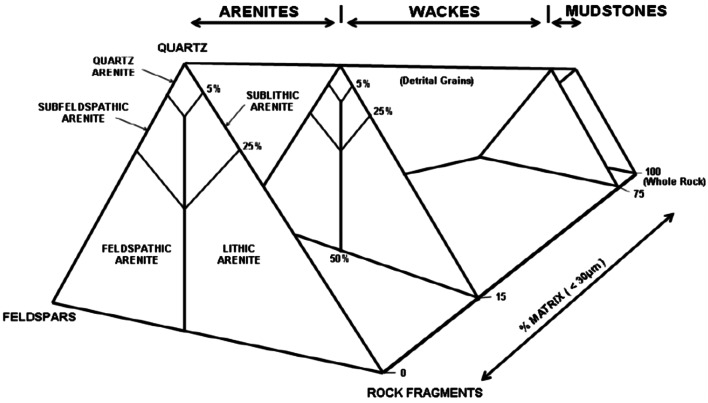
Sandstone classification diagram modified from^36,37^.

**Fig. 41:**
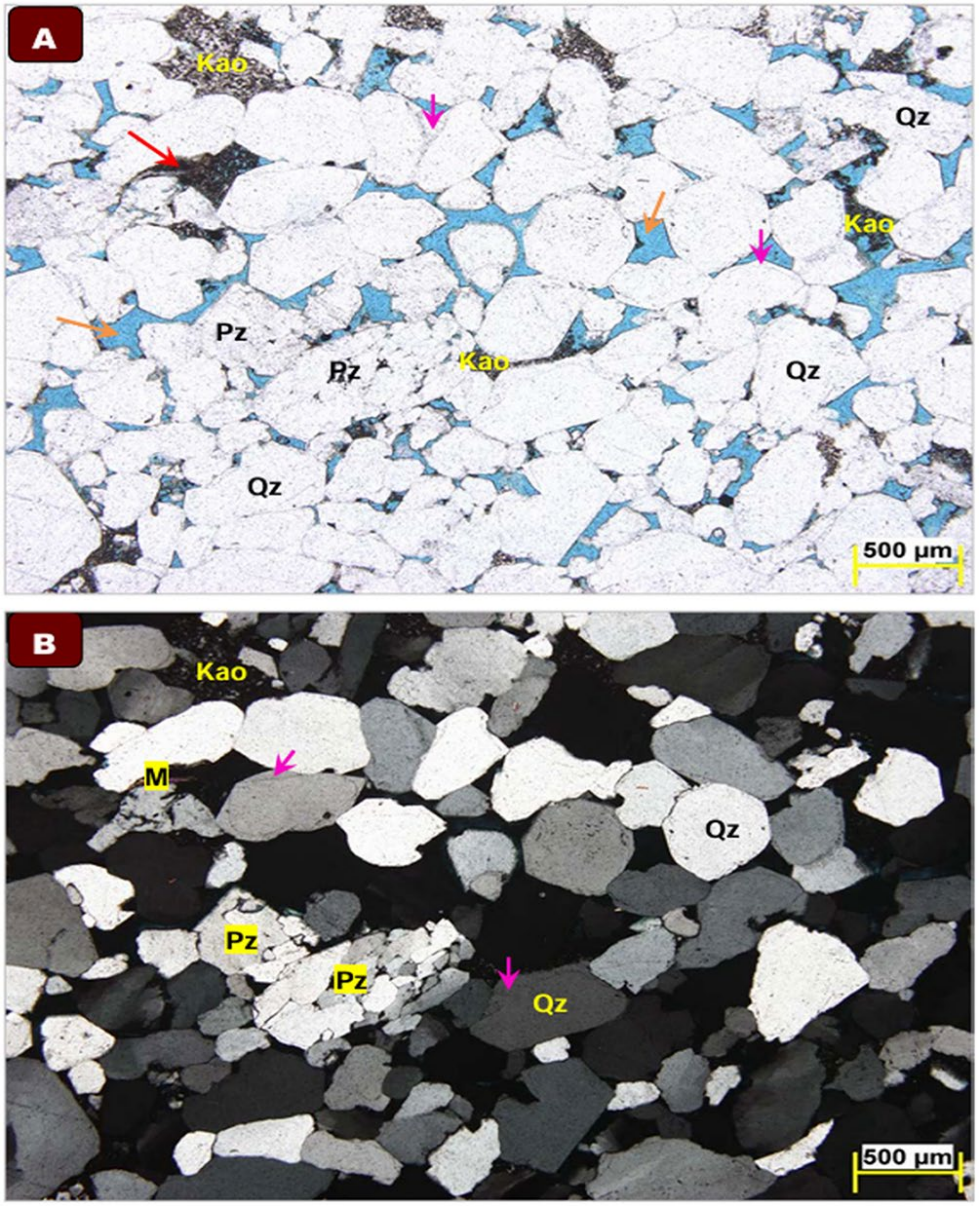
Pore filling with clay minerals (Kao) Kaolinite, (Qz) Quartz and (s) Siderite. The Two samples show quartz overgrowths for sample depth 11362 ft in (AEB-3D) of well PTAH-1X.

**Fig. 42:**
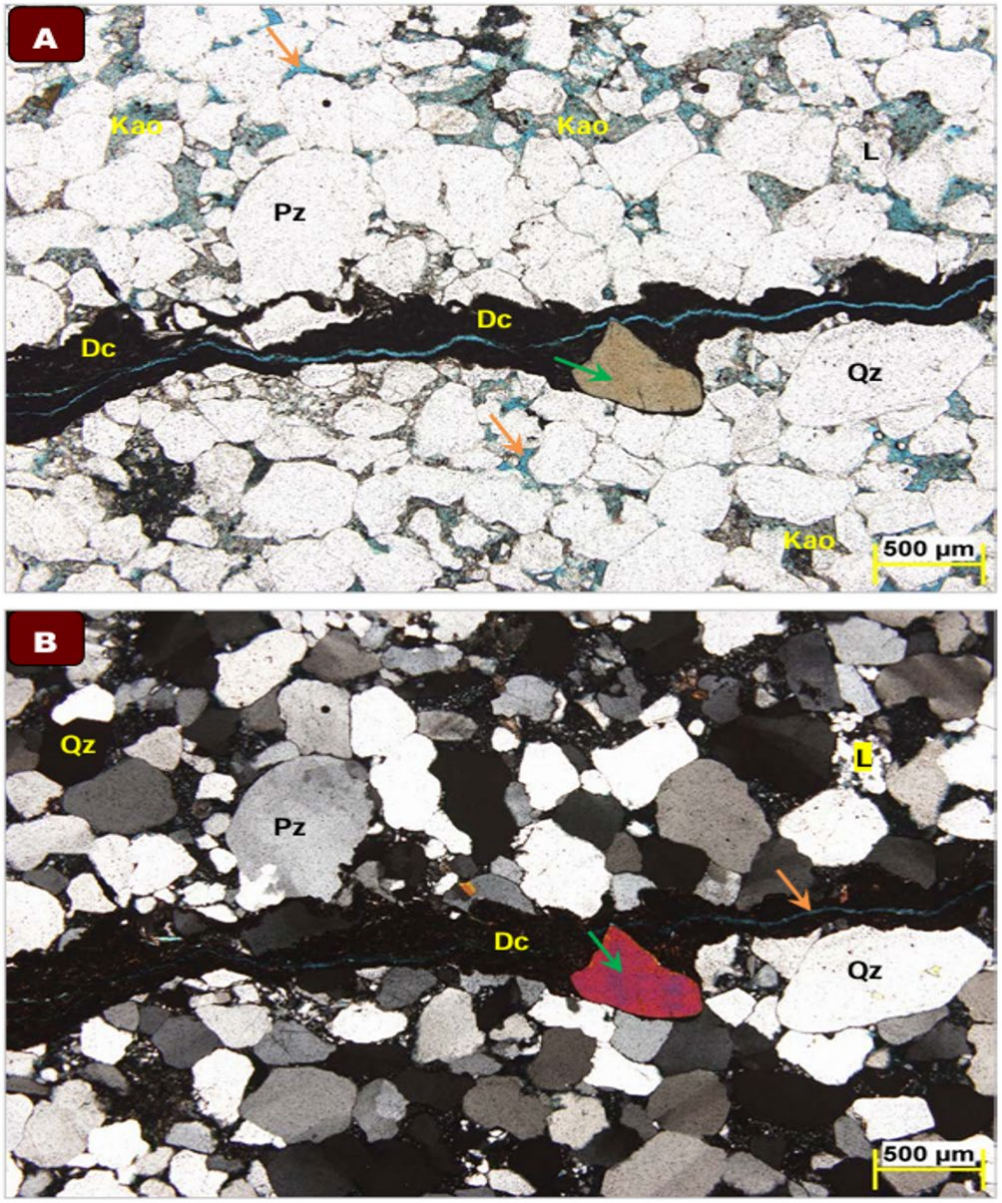
Pore filling with clay minerals (Kao) Kaolinite, (Qz) Quartz and Pz) Polycrystlline Quartz. The Two samples show quartz overgrowths for sample depth 12366 ft in (Shiffah B SD) of well PTAH-1X.

**Fig. 43:**
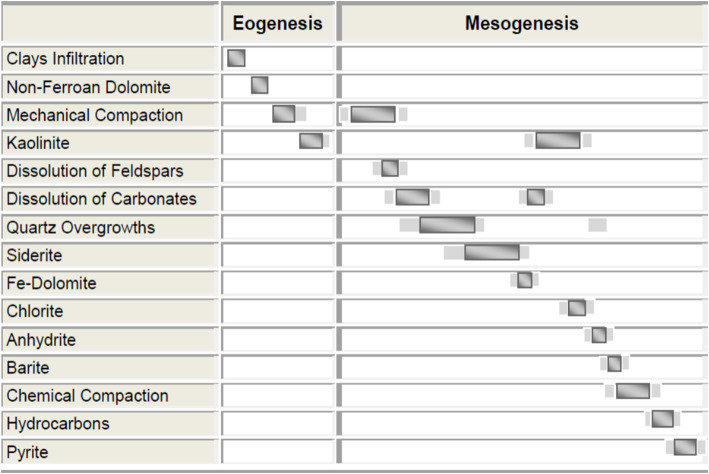
Paragenetic sequence diagram for the main diagenetic processes occurring in the sandstones of well PTAH-1X.

Petrographic classification identifies quartz-rich sandstones with minor feldspar and lithic fragments following standard sandstone classification schemes^36,37^ (Fig. 40; Table 6). Thin-section analysis (Figs. 41–42) reveals variable grain size, compaction textures, quartz overgrowths, and clay mineral occurrence. These features reflect heterogeneous pore geometry and cement distribution. The paragenetic sequence (Fig. 43) records progressive compaction, cementation, dissolution, and late mineral precipitation.

**Table 6:** **Petrographic analysis and rock names for PTAH-01X well based on**^36^.

**Well name**	**Sample TYPE**	**Sample No.**	**Formation**	**Depth MD (ft)**	**Rock Name (after Dott, 1964)**
PTAH-01X	Sidewall Core (SWC)	1	AEB-3D	11315	Quartz Arenite
		2	AEB-3D	11320	Sideritic Quartz Arenite
		3	AEB-3D	11327	Quartz Arenite
		4	AEB-3D	11362	Quartz Arenite
		5	AEB-3D	11373	Quartz Arenite
		6	AEB-3D	11378	Kaolinitic Quartz Arenite
		7	AEB-3E	11405	Kaolinitic Quartz Arenite
		8	AEB-3E	11415	Quartz Arenite
		14	Shiffah B SD	12333	Kaolinitic Quartz Arenite
		15	Shiffah B SD	12345	Quartz Arenite
		16	Shiffah B SD	12366	Quartz Arenite
		17	Shiffah B SD	12379	Quartz Arenite
		18	Shiffah B SD	12397	Quartz Arenite
		19	Shiffah B SD	12420	Kaolinitic Quartz Arenite
		20	Shiffah B SD	12452	Quartz Arenite
		21	Shiffah B SD	12476	Kaolinitic Quartz Arenite
		22	Shiffah B SD	12487	Kaolinitic Quartz Arenite
		23	Shiffah B SD	12500	Kaolinitic Quartz Arenite
		24	Shiffah B SD	12530	Quartz Arenite
		25	Shiffah B SD	12700	Quartz Arenite
		26	Shiffah B SD	12836	Kaolinitic Quartz Arenite
		27	Shiffah B SD	12944	Kaolinitic Quartz Arenite
		28	Shiffah B SD	13022	Quartz Arenite
		29	Shiffah C SH	13330	Kaolinitic Quartz Arenite
		30	Shiffah C SH	13420	Kaolinitic Quartz Arenite
		31	Shiffah C SH	13510	Kaolinitic Quartz Arenite

Core measurements from the PTAH-1X well (Table 7) indicate strong permeability anisotropy, with horizontal permeability exceeding vertical permeability, indicating directional variability in pore connectivity. This variability corresponds to differences in grain packing and cementation observed petrographically. Recorded permeability values range from (0.02 to 839 mD.), reflecting significant variability in reservoir properties. Porosity and fluid saturation data derived from core and log analysis show heterogeneous reservoir behavior. Intervals with lower shale content generally exhibit higher effective porosity and reduced water saturation, whereas shale-rich zones display lower pore connectivity and elevated water saturation. These trends demonstrate vertical variability in reservoir properties across the studied interval. Although petrographic observations are based on a limited number of samples, they provide representative insights into the dominant diagenetic processes affecting reservoir quality.

**Table 7:** Fluid saturation, porosity and permeability results for PTAH-01X well.

**Sample No.**	**Depth MD (ft)**	**Formation**	**Nitrogen permeability (mD)**	**Oil saturation (%)**	**Water saturation (%)**	**Helium porosity (%)**	**Grain density (gm/cc)**
1	11315	AEB-3D	146			21.1	2.67
2	11320	AEB-3D	11			16.6	2.71
3	11327	AEB-3D	52.8			19.8	2.66
4	11362	AEB-3D	94.3			19.5	2.65
5	11373	AEB-3D	58.8			16.6	2.66
6	11378	AEB-3D	14.1	5.7	57.6	15.6	2.66
7	11405	AEB-3E	3.61	11.5	48.1	11.3	2.64
8	11415	AEB-3E	100	8.8	42	11.9	2.65
17	12333	Shiffah B SD	34.4			15.8	2.65
18	12345	Shiffah B SD	611			17.5	2.68
19	12366	Shiffah B SD	120			14.4	2.65
20	12379	Shiffah B SD	70.1			16.2	2.65
21	12397	Shiffah B SD	128			16	2.65
22	12420	Shiffah B SD	157			16.1	2.65
23	12452	Shiffah B SD	412	1.3	45.3	17.1	2.65
24	12476	Shiffah B SD	46.6	3.3	43.2	13.8	2.64
25	12487	Shiffah B SD	45.7	1.2	47.6	16.5	2.65
26	12500	Shiffah B SD	104	4.2	42.5	14.5	2.65
27	12530	Shiffah B SD	199			17.5	2.65
28	12700	Shiffah B SD	839			15.3	2.66
29	12836	Shiffah B SD	330			17.2	2.65
30	12944	Shiffah B SD	14.1			15.1	2.65
31	13022	Shiffah B SD	177			15.9	5.66
38	13330	Shiffah C SH	126			16.5	2.66
39	13420	Shiffah C SH	20.1			11.7	2.66

### Integration and uncertainty considerations

Seismic, petrophysical, and petrographic datasets were integrated to establish relationships between structural framework and reservoir behavior. Structural mapping provided spatial

context for petrophysical trends, while petrographic observations supported interpretation of lithological controls. Uncertainty in the present study mainly arises from seismic resolution limitations, time-to-depth conversion, and spatial interpolation between wells. Structural interpretations near fault zones may be affected by seismic resolution and reflector continuity. The petrophysical parameter distribution maps may be influenced by the limited number of wells available for interpolation. In addition, the petrographic samples represent localized reservoir conditions and may not capture full field-scale heterogeneity. Despite these limitations, the overall spatial trends observed in reservoir properties and structural configurations remain consistent across independent datasets, supporting the reliability of the first-order interpretations.

## Discussion

The integrated seismic, structural, petrophysical, and petrographic results collectively demonstrate that hydrocarbon distribution in the Ptah Field is primarily governed by tectonic architecture interacting with lithological heterogeneity. The Shushan Basin is characterized by NW–SE trending normal faults forming horst and graben geometries. These fault systems control the structural configuration of the reservoirs and influence hydrocarbon migration and trapping within fault-bounded structural closures. Early Mesozoic extensional tectonics generated normal faults and horst–graben structures that influenced sediment distribution and created potential structural traps. Later tectonic activity associated with the Syrian Arc deformation led to partial fault reactivation and enhanced structural closure development. These tectonic processes-controlled hydrocarbon migration pathways and favored accumulation within structurally elevated fault blocks. Seismic interpretation reveals a fault-controlled framework characterized by extensional deformation and block segmentation that dictates reservoir preservation and trap geometry. Structurally elevated blocks consistently coincide with preserved reflector continuity and mapped closures, highlighting the dominant influence of fault geometry on reservoir organization. Structural mapping confirms vertical continuity of fault-controlled compartments across stratigraphic levels. The preservation of closures in the Bahariya, Alam El Bueib, and Paleozoic Shiffah B sand intervals indicates that tectonic positioning governs reservoir continuity and trapping efficiency.

An important structural–stratigraphic relationship highlighted by this study is the fault-controlled juxtaposition (Fig. 19) of the shale-dominated Upper Bahariya, Alam El Bueib-3C and Alam El Bueib-6 intervals against the Lower Bahariya, Alam El Bueib-3D and Paleozoic Shiffah B sandstone reservoirs. Normal fault displacement locally places low-permeability shale strata in direct contact with reservoir-quality sandstones, forming effective lateral sealing boundaries. This configuration promotes compartmentalization and contributes to the localized preservation of hydrocarbon accumulations within fault-bounded domains. Variations in hydrocarbon occurrence between structurally adjacent wells are therefore interpreted as a consequence of juxtaposition-controlled sealing rather than solely petrophysical variability. The spatial association between these juxtaposed contacts and mapped structural closures supports a tectono-stratigraphic trapping model in which sealing efficiency is enhanced by lithological contrast across fault planes. Within this framework, recently recognized accumulations are interpreted as structurally preserved compartments where favorable juxtaposition relationships improve trap integrity. Structural trapping appears to be the dominant mechanism controlling hydrocarbon accumulation, although minor stratigraphic or capillary influences cannot be completely excluded. Petrophysical trends reveal that shale distribution exerts a secondary but significant control on reservoir quality. Intervals characterized by lower shale volume exhibit higher effective porosity and reduced water saturation, whereas shale-rich zones display diminished pore connectivity. This relationship mirrors reservoir heterogeneity confirming that lithological variability modulates storage capacity within structurally defined compartments. Structural position, however, remains the primary control governing hydrocarbon presence. Petrographic analysis provides micro-scale evidence supporting these reservoir-scale relationships. Quartz-dominated sandstones with variable clay cementation exhibit heterogeneous pore structures that influence permeability anisotropy. The paragenetic evolution recorded in the core samples demonstrates how compaction and cementation modify pore geometry, reinforcing the link between lithological evolution and petrophysical variability within structurally preserved domains. Structural mapping indicates the presence of multiple fault-bounded structural compartments across the studied stratigraphic intervals, including several closures identified in the Bahariya, Alam El Bueib-3D and Paleozoic Shiffah B sand reservoirs. Variability in hydrocarbon occurrence is therefore interpreted as predictable tectono-stratigraphic behavior governed by fault geometry, sealing efficiency, and preservation history. Structurally elevated closures characterized by lower shale content and higher effective porosity represent the most favorable exploration targets, whereas deeper fault-bounded compartments and shale-rich intervals may involve relatively higher exploration risk. From an exploration perspective, the integration of structural mapping with petrophysical property distribution allows the identification of several structurally favourable zones within the study area. These zones correspond to fault-bounded structural highs where increased net pay thickness, higher effective porosity, and lower water saturation are observed. Such structurally elevated compartments represent promising targets for future drilling and reservoir development in the Ptah Field.

Overall, the integrated seismic, petrophysical, and petrographic interpretation demonstrates that hydrocarbon prospectivity in the Ptah Field is primarily governed by fault-controlled structural traps combined with lithological heterogeneity and juxtaposition-driven sealing. This integrated framework significantly improves the understanding of reservoir compartmentalization and reduces exploration uncertainty within the Shushan Basin. Despite the integrated approach applied in this study, some technical uncertainties remain. Future studies incorporating 3D seismic data, additional well data, and fault seal modeling would help to further refine the structural interpretation and reduce uncertainty in reservoir compartmentalization and hydrocarbon distribution.

## Conclusion

- The integrated workflow combining seismic interpretation, petrophysical analysis, and petrographic observations provides a transferable framework for evaluating fault-controlled clastic reservoirs in extensional basin settings.
- Hydrocarbon accumulation in the Ptah Field is primarily controlled by fault-related structural traps associated with extensional tectonics and subsequent fault reactivation, where structurally elevated horst blocks and tilted fault domains represent the most favorable trapping configurations.
- The Paleozoic Shiffah B SD, Alam El Bueib (AEB-3D/3E) and Bahariya reservoirs constitute the main productive intervals due to their structural positioning and sealing relationships.
- Reservoir quality alone does not govern hydrocarbon occurrence; effective accumulation requires favorable tectono-stratigraphic conditions.
- The Paleozoic Shiffah B sand reservoir exhibits spatial discontinuity associated with tectonic uplift and erosional truncation, while fault segmentation produces reservoir compartmentalization that contributes to variability in hydrocarbon distribution.
- Reservoir quality within the Paleozoic Shiffah B SD and Alam El Bueib (AEB-3D/3E) intervals shows moderate variability, with effective porosity values reaching up to ~18–21% and permeability values ranging up to ~839 mD, reflecting heterogeneous pore systems within the studied reservoirs.
- This integrated interpretation highlights several structurally elevated fault-bounded closures that represent promising targets for future exploration drilling within the Ptah Field.
- Although the integrated interpretation provides a reliable structural framework, some uncertainty remains due to seismic resolution, structural complexity of the fault system, and limited well control within the study area.

## Data Availability

The data supporting the conclusions of this investigation are accessible upon request from the corresponding author. Due to privacy and ethical concerns, the data is not made public.
